# New Insights into the Structure and Thermodynamic Stability of Polymorphs I and II of the Nicotinamide:Adipic Acid Co-Crystal: A BEST-CSP Study

**DOI:** 10.1021/acs.cgd.6c00576

**Published:** 2026-06-23

**Authors:** Inês O. Feliciano, Carlos E. S. Bernardes, M. Fátima M. Piedade, M. Soledade C. S. Santos, Paolo P. Mazzeo, Jan Blahut, Martin Dračínský, Manuel E. Minas da Piedade

**Affiliations:** † Centro de Química Estrutural, Institute of Molecular Sciences, Departamento de Química e Bioquímica, Faculdade de Ciências, 37809 Universidade de Lisboa, 1749-016 Lisboa, Portugal; ‡ Department of Chemical Science, Live Science and Environmental Sustainability, 9370 University of Parma, Parco Area delle Scienze 17/A, 43124 Parma, Italy; § Institute of Organic Chemistry and Biochemistry, Czech Academy of Science, Flemingovo nám. 2, 160 00 Prague, Czech Republic

## Abstract

The polymorphism of the 1:1 nicotinamide:adipic acid (NIC:AA) cocrystal system was re-examined by combining structural and thermodynamic approaches. Differential scanning calorimetry (DSC), solid-state NMR (ssNMR), and variable-temperature powder X-ray diffraction (VT-PXRD) confirmed an enantiotropic relationship between the two polymorphs, evidenced by a direct solid–solid transformation from triclinic form I to monoclinic form II at ∼ 370 K. Solubility data subsequently indicated that this temperature is ∼78 K higher than the equilibrium transition temperature (289.2 ± 0.7 K), consistent with a phase transition hindered by a substantial activation barrier. This also explained why the reverse process was not detected on cooling within the time scale of the DSC, ssNMR, and VT-PXRD experiments. The relative stabilities of forms I and II, as well as their stability relative to dissociation into the coformers, at 298 K, were quantitatively established based on Gibbs energy, enthalpy, and entropy data obtained from solution calorimetry and solubility measurements. The results indicated that at 298 K: (*i*) form II is thermodynamically more stable than form I, despite having a lower lattice enthalpy; (*ii*) this is consistent with the conclusion that the equilibrium transition temperature (289.2 ± 0.7 K) is lower than 298 K and suggests that the stabilization of form II (the high-temperature polymorph) versus form I (low-temperature polymorph) under ambient conditions is of an entropic nature; (*iii*) both polymorphs are stable with respect to dissociation into the coformers nicotinamide and adipic acid; and (*iv*) the stability gain upon cocrystallization is primarily of an enthalpic rather than entropic nature, reflecting a lattice enthalpy advantage relative to the pure components. Single-crystal X-ray diffraction further showed that at ∼298 K form I exhibits an ∼3% higher density and packing index than form II, in line with the observed lattice enthalpy difference Δ_lat_ *H*_m_^o^(crI) > Δ_lat_ *H*_m_^o^(crII). Finally, although cocrystallization is frequently used to produce solid forms with enhanced solubility or stability relative to the individual coformers, the formation of the NIC:AA cocrystal does lead to improved solid-state stability but not to solubility enhancement, at least when acetonitrile is used as solvent.

## Graphical abstract

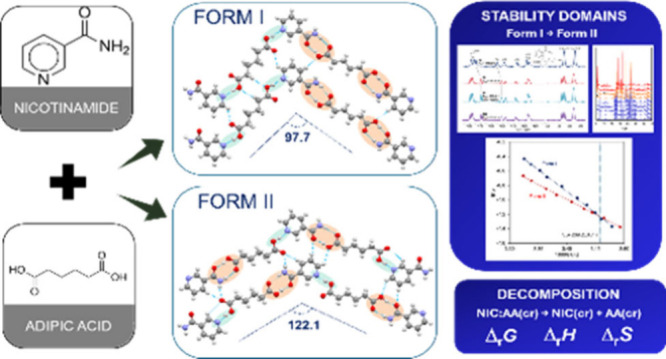

## Introduction

Initially discovered more than a century ago,^1^ cocrystallization has been, in recent years, one of the leading strategies to expand the solid form landscape and tune the physicochemical properties of functional organic materials.^2,3^ It was once considered that the use of cocrystals could not only provide opportunity for tunning the efficacy of an “active” molecule through selection from a larger number of crystal forms, but also constituted an effective way to avoid polymorphism and the associated complications for manufacturing products with highly reproducible properties.^4^ This last conception was rooted on the idea that the noncovalent interactions holding different molecules in a crystal lattice could require more specific packing modes and hinder polymorphic transformations.^5^ However, as the number of cocrystals being reported increased, it became apparent that polymorphism was also common in these multicomponent systems.^4,6,7^ Similarly to single component crystals, distinct cocrystal polymorphs can display significantly different properties. Thus, strict control over polymorphism is also a *sine qua non* condition for the preparation of cocrystal-based products with highly reproducible properties and function.

Different cocrystal polymorphs can often coexist under the same pressure–temperature conditions, but only one will be thermodynamically stable. In the absence of sufficiently high kinetic barriers metastability cannot be sustained, and all metastable polymorphs will tend to evolve over time to the thermodynamically stable form. Therefore, once cocrystal polymorphism has been identified, structural characterization should be accompanied by a thorough assessment of the relative stability of the different forms in the temperature range of interest for the application in view. An important progress within this scope has been the combination of experimental and *in silico* polymorph screenings. Crystal structure prediction (CSP) methodologies have become of great interest to forecast the stability hierarchy of plausible crystal forms of a given compound. These predictions can considerably help the assessment of manufacture risks associated with polymorphism, thus contributing to avoid the occurrence of serious incidents, as showcased by the ritonavir example.^8^ CSP methods have advanced considerably in recent years, and applications have already covered cocrystals.^9–11^ Their development requires, however, accurate thermodynamic data (e.g., lattice energies; temperatures, enthalpies, entropies, and Gibbs energies of polymorphic phase transitions or cocrystal dissociation into the precursors) that can be used to benchmark the predictions of structural landscapes and expand potential applications to, for example, the forecast of thermal stability domains or solubilities of different polymorphs.

Experimental Gibbs energy, enthalpy and entropy data for cocrystal dissociation into the precursors are scarce, representative examples being quinidrone,^12^ nicotinamide:mandelic acid,^13^ felodipine:4,4’-bipyridine,^14^ carbamazepine:saccharin,^15^ diflunisal:nicotinamide,^16^ melatonin:pimelic acid,^17^ theophylline:salicylic acid,^18^ celecoxib:nicotinamide,^19^ fumaric acid:glycine,^20^ maleic acid:glycine,^20^ maleic acid: l-phenylalanine,^21^ and carbamazepine:benzamide.^22^ Similar data for polymorphic phase transitions of bi- and multicomponent crystals are even scarcer, and in most cases the performed experiments provide only information on the temperature and enthalpy of the phase transition with a reliability that is impossible to assess. Representative examples include the nicotinamide:(*R*)-mandelic acid,^23^ carbamazepine:nicotinamide,^24^ and metronidazole benzoate:salicylic acid systems.^25^ Addressing this gap is a central goal of COST Action BEST-CSP (Bringing Experiment and Simulation Together in Crystal Structure Prediction), an European initiative combining efforts from experts on CSP and experimentalists working on the determination accurate structural, thermodynamic, and physical property data for a number of model systems, against which the theoretical predictions may be tested.^26^

The present study is part of this effort. It is focused on the nicotinamide (NIC):adipic acid (AA) cocrystal system (Figure 1), which is known to crystallize in two polymorphic forms of 1:1 stoichiometry: the triclinic form I^27^ (CSD ref code NUKYIC),^28^ and the monoclinic form II^29^ (CSD ref code NUKYIC01).^28^ Based on calorimetric and solubility measurements the relative stability of both forms, under ambient temperature (298 K) and pressure (1 bar) has been established, on Gibbs energy, enthalpy, and entropy grounds. The experiments also afforded stability relative to decomposition into the precursors. Finally possible structure–stability links were discussed, by combining thermodynamic, X-ray diffraction (single crystal and powder), and solid-state nuclear magnetic resonance information.

**Figure 1:**
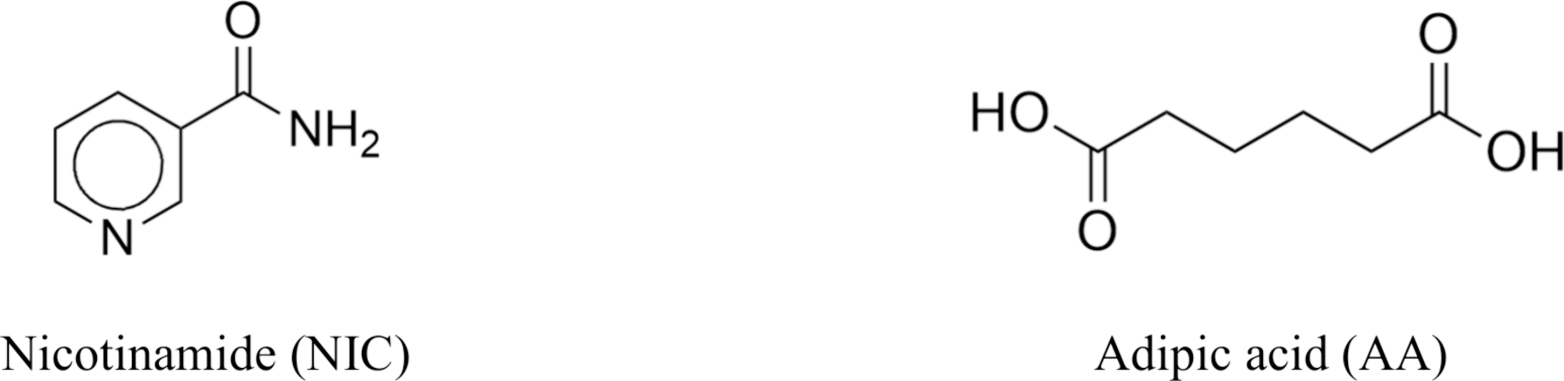
Molecular structures of the two coformers used in this work.

## Materials and Methods

### Materials

#### Solvents

DMSO (Thermo Scientific, HPLC grade, 99.9%), acetonitrile (Honywell, > 99.9%), methanol (Sigma-Aldrich, HPLC grade, > 99.9%), chloroform (Lab-Scan, 99.5%), and ethanol (Carlo Erba, HPLC grade, > 99.9%) were used as received.

#### Starting Materials

Nicotinamide (NIC, Acros Organics, 99.0%, CAS Nr 98-92-0) was used without further purification. Elemental analysis for C_6_H_6_N_2_O (molar percentage; mean of two determinations): expected C 59.0%, N 22.9%, H 5.0%; found C 58.6 ± 0.3%, N 22.7 ± 0.3%, H 5.1 ± 0.1%. The PXRD pattern obtained at 296 ± 2 K for NIC was indexed as monoclinic, *P*2_1_/*c*, *a* = 3.972(4) Å, *b* = 15.636(6) Å, *c* = 9.418(9) Å, β = 99.06(20)° in good agreement with the single crystal X-ray diffraction data (SCXRD) reported for polymorph I (CSD RefCode: NICOAM05):^28,30^ monoclinic, *P*2_1_/*c*, *a* = 3.97447(1) Å, *b* = 15.6420(7) Å, *c* = 9.4298(5) Å, β = 99.0234(29)°. Adipic acid (AA, C_6_H_10_O_4_, TCI, 99.9%, CAS Nr 124–04–9) was used as received. Elemental analysis for C_6_H_10_O_4_: expected C 49.3%, H 6.9%; found C 48.8 ± 0.3%, H 6.9 ± 0.1%; PXRD pattern obtained at 296 ± 2 K was indexed as monoclinic, *P*2_1_/*n*, *a* = 7.364(10) Å, *b* = 5.178(11) Å, *c* = 10.029(11) Å, β = 110.66(40)°. This indicated that the sample corresponded to polymorph I (CSD RefCode: ADIPAC14):^28,31^ monoclinic, *P*2_1_/*n*, *a* = 7.3738(17) Å, *b* = 5.1520(9) Å, *c* = 10.016(2) Å, β = 110.47(2)°.

#### Co-Crystal Syntheses

Form I of the nicotinamide:adipic acid (NIC:AA) cocrystal was prepared by mechanochemistry. Equimolar amounts (0.37 mmol; 100 mg of mixture) of nicotinamide and adipic acid were milled for 20 min, at 30 Hz, in a 10 cm^3^ stainless-steel jar, with two 10 mm diameter stainless steel balls. The powder pattern of the obtained product, acquired at 296 ± 2 K, was indexed as triclinic, *P* 1, *a* = 5.050(2) Å, *b* = 5.397(2) Å, *c* = 24.236(10) Å, α = 93.82(3)°, β = 93.66(3)°, γ = 90.99(4)°. These values are in excellent agreement with the single crystal X-ray diffraction (SCXRD) results obtained in this work for form I, at 298 ± 2 K (see below): triclinic, *P* 1, *a* = 5.0516(15) Å, *b* = 5.3999(17) Å, *c* = 24.260(8) Å, α = 93.996(12)°, β = 93.766(12)°, γ = 90.933(12)°.

The synthesis of the nicotinamide:adipic acid form II polymorph was identical to that described for form I, except that 15 μL of methanol were added to the jar. The powder pattern of the obtained product, acquired at 296 ± 2 K, was indexed as monoclinic, *P*2_1_/*c*, *a* = 4.978(5), *b* = 30.268(23) Å, *c* = 8.935(8) Å, β = 93.68(27)°. These values are in good agreement with the SCXRD results obtained in this work for form II, at 298 ± 2 K (see below), *P*2_1_/*c*, *a* = 4.981(5) Å, *b* = 30.35(3) Å, *c* = 8.932(8) Å, β = 93.76(3)° and previously reported at 295 K (CSD RefCode: NUKYIC01),^28,29^ monoclinic, *P*2_1_/*c*, *a* = 4.9815(2) Å, *b* = 30.3259(19) Å, *c* = 8.9413(4) Å, β = 93.776(4)°.

### Elemental Analysis

Elemental C, H, and N analyses were performed on a Fison Instruments *EA* apparatus, with a typical accuracy error of ± 0.3% for carbon and nitrogen and ±0.1% for hydrogen. These uncertainties are based on interlaboratory tests carried out between national certified laboratories and the ISO 11352/2012 standards.

### Powder X-ray Diffraction (PXRD)

Powder X-ray diffraction analyses used in the identification of the crystal forms studied in this work were carried out at 296 ± 2 K, on a Philips X’Pert PRO X-ray diffractometer, operating in the θ-2θ mode. The apparatus was equipped with a PW 3050/60 vertical goniometer and an X’Celerator detector. The radiation was produced with a Cu–Kα (λ = 1.5406 Å) tube, operated at 40 kV and 30 mA. Data were collected in the 5–35° 2θ range, with a step size of 0.017°, and a step duration of 40 s. Aluminum sample holders were used. The indexation of the powder patterns was performed with CelRef.^32^

### Variable Temperature Powder X-ray Diffraction (VT-PXRD)

VT-PXRD experiments were performed on both NIC:AA polymorphs, using a Rigaku SmartLab XE diffractometer, equipped with a solid-state Hypix3000 2D detector, and an Anton-Paar TTK600 nonambient chamber. The samples were placed on a flat copper holder and heated in steps, at ambient pressure, holding the sample for thermalization for 1 min before each data acquisition. Data was collected at the following temperatures: 297.9, 313.1, 328.1, 343.1, 358.1, 368.1, 378.1, 383.1, and 388.1 K, for form I; 298.8, 313.1, 328.1, 343.1, 358.1, and 373.1 K, for form II. The powder patterns were collected in 1D mode, in the 5–40° 2θ range, with a scan rate of 10° min^–1^. A length-limiting slit of 10 mm, compatible with the nonambient chamber, was used to enhance X-ray flux over the sample, while 2.5° soller slits were used to improve the peak profile and limit the overlapping of reflections. Quantitative phase analysis as well as unit cell parameters as a function of temperature were obtained by Rietveld refinement, using TOPAS v6 software.^33^

### Single-Crystal X-ray Diffraction (SCXRD)

Crystals of NIC:AA form I suitable for single crystal X-ray diffraction analysis were obtained at ambient conditions (298 ± 2 K; ∼ 1 bar) by solvent evaporation from a saturated solution prepared using the mechanochemical product and a mixture of chloroform (10 mL) and methanol (50 μL). Form II crystals were prepared by heating form I powder above the temperature of the I → II solid–solid phase transition, followed by cooling to room temperature. Bruker D8 Venture and Bruker AXS-KAPPA APEX II diffractometers were used in the study of forms I and II, respectively. Both diffractometers were equipped with monochromated Mo–Kα (λ = 0.71073 Å) radiation sources, operating at 50 kV and 30 mA. The crystals were coated with Paratone-N oil and mounted on a Kapton loop. Data collection was monitored with the Bruker APEX IV software.^34^ An empirical absorption correction was enforced using Bruker SADAB^35^ and data reduction was done with Bruker SAINT.^36^ The structures were solved by intrinsic phasing with Bruker SHELXT-2017 ^37^ and refined by full-matrix-least-squares on *F* ^2^ using SHELXL-2017,^38^ both programs included in WINGX-Version 2018.3.^39^ The atoms were refined with anisotropic thermal parameters. Most of the hydrogen atoms were inserted in calculated positions and allowed to refine riding on the parent carbon atom. A summary of the crystal data, structure solution, and refinement parameters is given in the Supporting Information (Table S7). Structural representations were prepared using Mercury 2025.1.1.^40^

### Solid-State Nuclear Magnetic Resonance (ssNMR)

The ssNMR experiments were carried out on a JEOL 600 MHz spectrometer, equipped with a 3.2 mm HX probe, at a 14.1 T field corresponding to a 600 MHz ^1^H Larmor frequency. The sample was packed in a 3.2 mm zirconia rotor. The ^13^C­{^1^H} spectra were acquired using a cross-polarization experiment with 5 ms contact time and preacquisition delay set to 1.5 × *T*_1_(^1^H) (as obtained from ^1^H saturation recovery experiment). The number of coadded transients ranged from 16 to 256 depending on the sensitivity of the sample at the selected temperature. The chemical shift of the adamantane CH resonance at 37.8 ppm was used as an external secondary reference for calibration of the ^13^C chemical-shift scale. The ^1^H and ^13^C nutation frequencies were 73.3 kHz and 54.1 kHz, respectively. Real sample temperature (including MAS frictional heating) was determined using the chemical shift of ^207^Pb.^41^ All raw data (^13^C, ^1^H spectra and ^1^H saturation recovery experiments) is available in Zenodo.^42^

NMR chemical shift calculations were carried out using the Gauge Including Projector Augmented Wave (GIPAW) method under periodic boundary conditions,^43,44^ as implemented in the CASTEP software (version 22.11).^45^ CASTEP operates within a density functional theory (DFT) framework, employing pseudopotentials to account for core–electron effects and plane-wave basis sets to represent valence electrons. Before the NMR calculations, the atomic positions within the experimental unit cell were geometry-optimized while keeping the unit-cell parameters fixed. Both the geometry optimization and NMR calculations were performed using the Perdew–Burke–Ernzerhof (PBE) exchange-correlation functional,^46^ with a plane-wave energy cutoff of 600 eV. Default ultrasoft, on-the-fly generated pseudopotentials^47^ were used throughout. Brillouin zone sampling was conducted using a Monkhorst–Pack *k*-point grid^48^ with a minimum spacing of 0.1 Å^–1^, and empirical dispersion interactions were accounted for using the Tkatchenko–Scheffler (TS) correction scheme.^49^

### Differential Scanning Calorimetry (DSC)

DSC experiments were performed on a PerkinElmer DSC-7. The apparatus was controlled by the PerkinElmer Pyris V. 7.0.0.0110 software, which was also used for data acquisition and analysis. The temperature range and heating rate were 298–435 K and 5 K min^–1^, respectively. The samples with 1.5–3.5 mg mass were contained in 20 μL aluminum pans (PerkinElmer 0219-0062), hermetically sealed in air. Weighing with a precision of ±0.1 μg was done on a Mettler XP2U ultramicro balance. The purging gas was nitrogen (Praxair 5.0, 99.999%) at a flow rate of 30 cm^3^ min^–1^. The temperature scale of the apparatus was calibrated at the heating rate of the sample measurements using the following standards: benzoic acid (NIST SRM 39j, 99.9996, *T*_fus_ = 395.50 K), indium (PerkinElmer, 99.999%, *T*_fus_ = 429.75 K, Δ_fus_ *h* = 28.45 J·g^–1^), lead (GoodFellow, 99.999, *T*_fus_ = 600.61 K), and zinc (PerkinElmer, 99.999%, *T*_fus_ = 692.65 K). The calibration of the heat flow scale was based on the standard specific enthalpy of fusion of the indium standard.

### Solubility Measurements

Dynamic solubility measurements in acetonitrile were performed with the in-house developed apparatus illustrated in Figure 2. The system was composed of an aluminum block, where a 3 cm^3^ glass vial containing the sample and solvent was inserted. The temperature of the block was measured with a RS Pro Pt100 sensor and controlled using a Peltier element (European Thermodynamics GM250-127-14-16), connected to Adafruit MAX31865 and Pololu High-Power Motor Driver 18v15 boards, respectively. The temperature sensor and the motor driver are linked to an Arduino Uno microcontroller, programmed with a PID software specifically designed for this device. This system allows the temperature of the aluminum block to be maintained or increased/decreased at a constant rate, with a precision of ±0.03 K. A white LED illuminates the solution through a hole situated below the sample vessel and the presence or absence of crystals in suspension was simultaneously monitored by two methods: (*i*) a USB Digital Microscope Zoom camera, which captures images of the solution every 1 s and (*ii*) an Advanced Photonix photocell NSL-19M51 sensor (also connected to the Arduino microcontroller) that measures the turbidity of the solution. The temperature of the sample under study was monitored with a second Pt100 probe (TE Connectivity, NB-PTCO-160) inserted in a glass well containing silicon oil, immersed in the solvent. The two Pt100 sensors used in the apparatus were calibrated against a reference Pt100 sensor, which had been previously calibrated at an accredited facility according to the ITS-90 temperature scale. The calibration was performed with the three sensors immersed in water inside the glass vial show in Figure 2. The results indicated that the precision in the measurements of the block and sample temperatures was ± 0.01 K.

**Figure 2:**
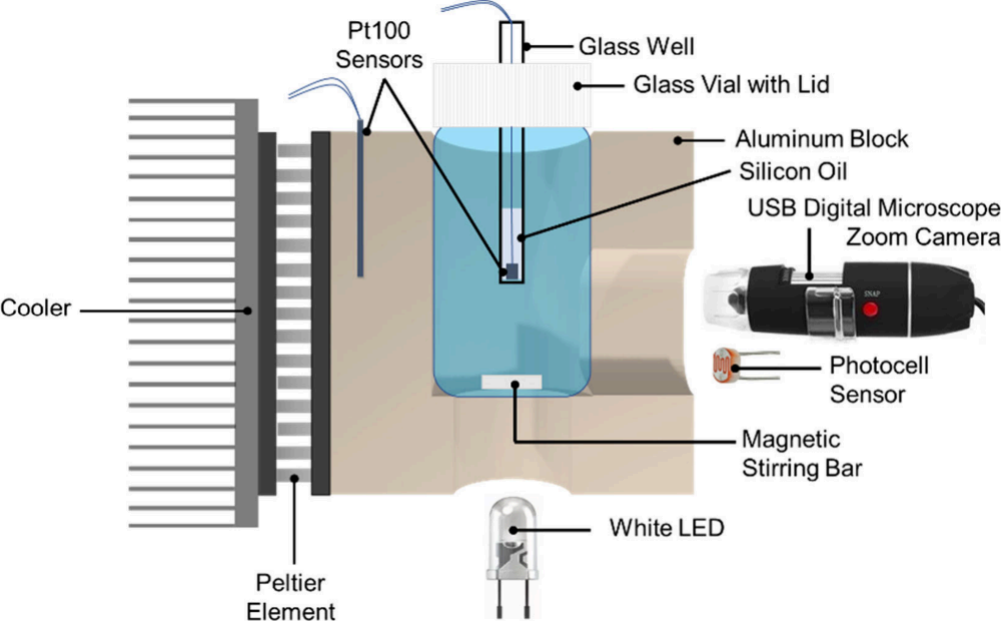
Apparatus used for solubility determinations from dynamic clear point measurements.

In a typical experiment, 5–50 mg of compound and ∼ 1.6 g of acetonitrile (previously placed in a refrigerator at 277 K) were weighed into the glass vial, with a precision of ± 0.01 mg, using a Mettler Toledo XS205 balance. The vial containing the suspension was transferred to the aluminum block, initially kept at 283 K. The sample was subsequently heated at 0.3 K min^–1^, under magnetic stirring, until complete dissolution of the solid was detected (clear point). The clear point temperature was taken as the average result of the imaging and turbidity measurements. The composition of the mixture was chosen to observe solubilization between 285 and 310 K.

### Solution Calorimetry

Enthalpies of solution in DMSO, at 298.15 K, were determined using a previously reported 15.0 cm^3^ stainless steel cell adapted to a LKB 2277 Thermal Activity Monitor (TAM).^50^ The cell was equipped with a sample holder, sample drop mechanism, stirrer, and electrical calibration system. In a typical experiment, 10–25 mg of sample, gently pressed into a pellet, was weighed with a precision of ± 0.1 μg on a Mettler XP2U ultramicro balance and transferred to the drop chamber of the calorimetric cell. The cell body, containing ∼ 14 g of solvent or solution, was weighed on a Mettler XS205 balance (±10 μg precision) and adjusted to the cell head. The assembled setup was transferred to the calorimeter, and stirring was initiated at 80 rpm. After thermal equilibration and acquisition of the initial baseline, the sample was dropped into the solvent/solution to start the dissolution process. The observed calorimetric curve was monitored until the signal returned to the baseline. The corresponding standard molar enthalpy was calculated from

1 $$\Delta_{\mathrm{sol}}H_{m}^{o}=\frac{M}{m}\varepsilon \left(A-A_{b}\right)$$

where *A* is the area of the measured curve; *m* and *M* are the mass and molar mass of the sample, respectively; *A*_b_ is the area correction associated with the drop process (i.e., opening the drop chamber without a sample inside), which was determined in independent experiments; and *ε* is the calibration constant of the calorimeter. The value of *ε* was obtained from a series of electrical calibrations, where a potential difference, *V*, was applied to a 20 Ω resistance inside the calorimetric cell, during a predetermined time, *t*. This caused a current of intensity, *I*, to flow through the resistance, leading to the dissipation of an amount of heat *Q* = *VIt*. The calculation of *ε* was based on *ε* = *Q*/*A*_c_, where *A*_c_ is the area of the calibration curve. Instrument control and data acquisition were performed using CBCAL 3.0.^51^ Data analysis was carried out with EasyGraph II.^52^ The accuracy of the electrical calibration had been previously found to be better than 0.06% based on a comparison of the experimentally determined enthalpy of solution of KCl in water (Δ_sol_ *H*_m_^o^ = 17.40 ± 0.06 kJ·mol^–1^; KCl:4115H_2_O final solution; mean of 10 independent determinations)^50^ and the corresponding value recommended in the NBS Tables (Δ_sol_ *H*_m_^o^ = 17.41 ± 0.11 kJ·mol^–1^).^53^

## Results and Discussion

All molar thermodynamic quantities were calculated based on the molar masses *M*(NIC:AA) = 268.269 g mol^–1^, *M*(NIC) = 122.127 g mol^–1^, and *M*(AA) = 146.142 g mol^–1^, obtained from the 2021 standard atomic masses recommended by the IUPAC Commission.^54^ The procedures used in the assignment of uncertainties to the lattice parameters determined by powder and single crystal X-ray diffraction and to all thermodynamic quantities are indicated in the Supporting Information.

### Structure

Single-crystal X-ray diffraction structures of NIC:AA have been reported at 180 K for form I (triclinic, CSD RefCode: NUKYIC)^27,28^ and at 295 K for form II (monoclinic, CSD RefCode: NUKYIC01).^28,29^ The structure of form I was redetermined here, at 298 ± 2 K, to allow direct structural comparison of the two polymorphs at a temperature compatible with the thermodynamic data discussed below, which refer to 298.15 K. For the sake of consistency, the structure of form II was also redetermined at 298 ± 2 K. A summary of the obtained crystal data (full details are given in the Supporting Information) and of the corresponding literature information is given in Table 1.

**Table 1:** Crystal Data for the Two NIC:AA Polymorphs

	Form I^a^	Form I	Form II^b^	Form II
Refcode or CCDC Nr.	NUKYIC	This work (CCDC 2535150)^c^	NUKYIC01	This work (CCDC 2535152)^c^
*T*/K	180	298(2)	295	298(2)
Crystal system	Triclinic	Triclinic	Monoclinic	Monoclinic
Space Group	*P* 1	*P* 1	*P*2_1_/*c*	*P*2_1_/*c*
*a*/Å	5.0110(1)	5.0516(15)	4.9815(2)	4.981(5)
*b*/Å	5.3379(1)	5.3999(17)	30.3259(19)	30.35(3)
*c*/Å	24.0348(6)	24.260(8)	8.9413(4)	8.932(8)
α/°	93.036(1)	93.996(12)	90	90
β/°	92.753(1)	93.766(12)	93.776(4)	93.76(3)
γ/°	92.189(1)	90.933(12)	90	90
*V*/Å^3^	640.71(2)	658.59(36)	1347.82(12)	1347(2)
*Z/Z’*	2/1	2/1	4/1	4/1
ρ_calc_/g cm^–3^	1.39055(4)	1.3528(7)	1.3221(1)	1.323
*k* ^d^	0.742	0.721	0.688	0.694

a References 27, 28 .

b References 28, 29 .

c CCDC number.

d Values of Kitaigorodskii’s packing index, *k*, were given by Mercury 2025.1.1.^40^

No significant differences were noted between the structural organization of form I at 180 K and 298 ± 2 K (Figure S1, Supporting Information), except for the thermal expansion of the unit cell. The structure of form II here determined at 298 ± 2 K is also in agreement with that previously reported at 295 K (CSD RefCode: NUKYIC01).^28,29^ The discussion will, therefore, be focused on the structures of forms I and II at ambient temperature, whose main packing features are illustrated in Figures 3 and 4.

**Figure 3:**
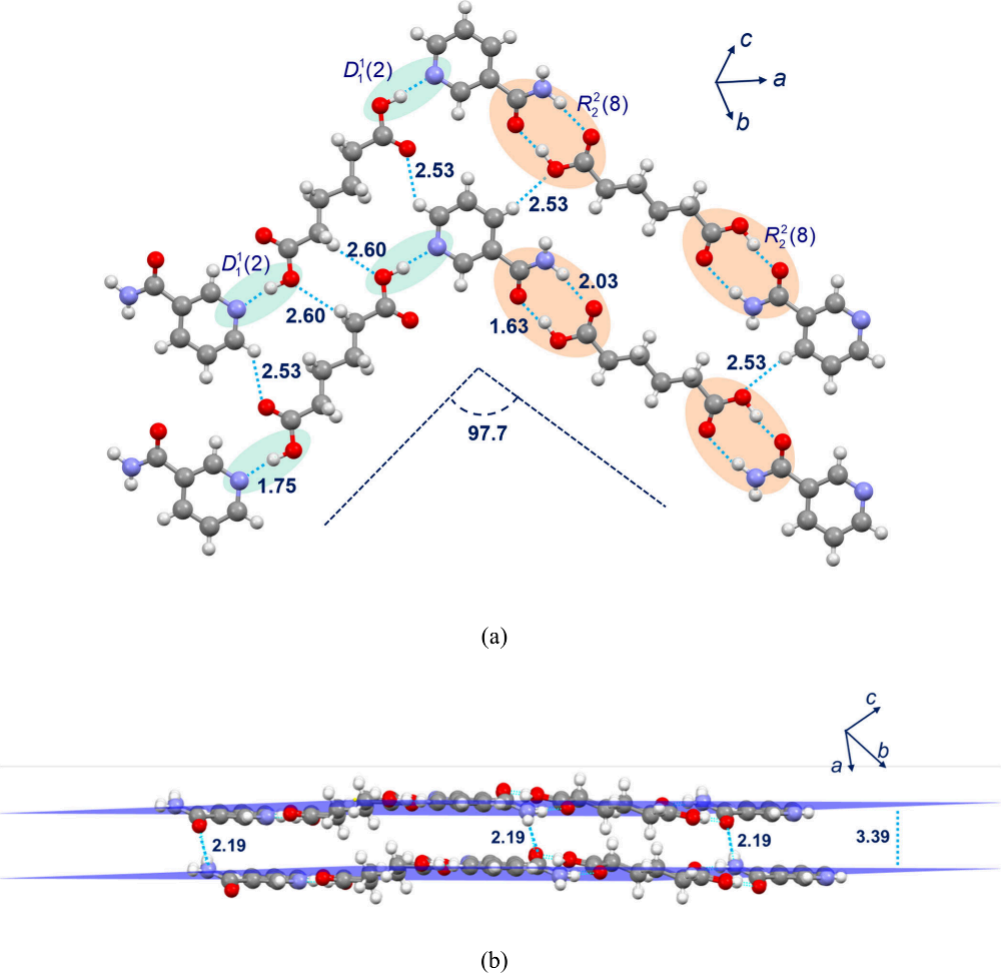
Crystalline structure of NIC:AA form I at 298 ± 2 K (distances in Å): (a) 1D chains sustained by *R*_2_^2^(8) and *D*_1_^1^(2) motifs and 2D sheets resulting from their interaction through nonclassical C–H···O hydrogen bonds; (b) separation between the layers of 2D sheets forming the 3D packing and intersheet NH···O hydrogen bond.

**Figure 4:**
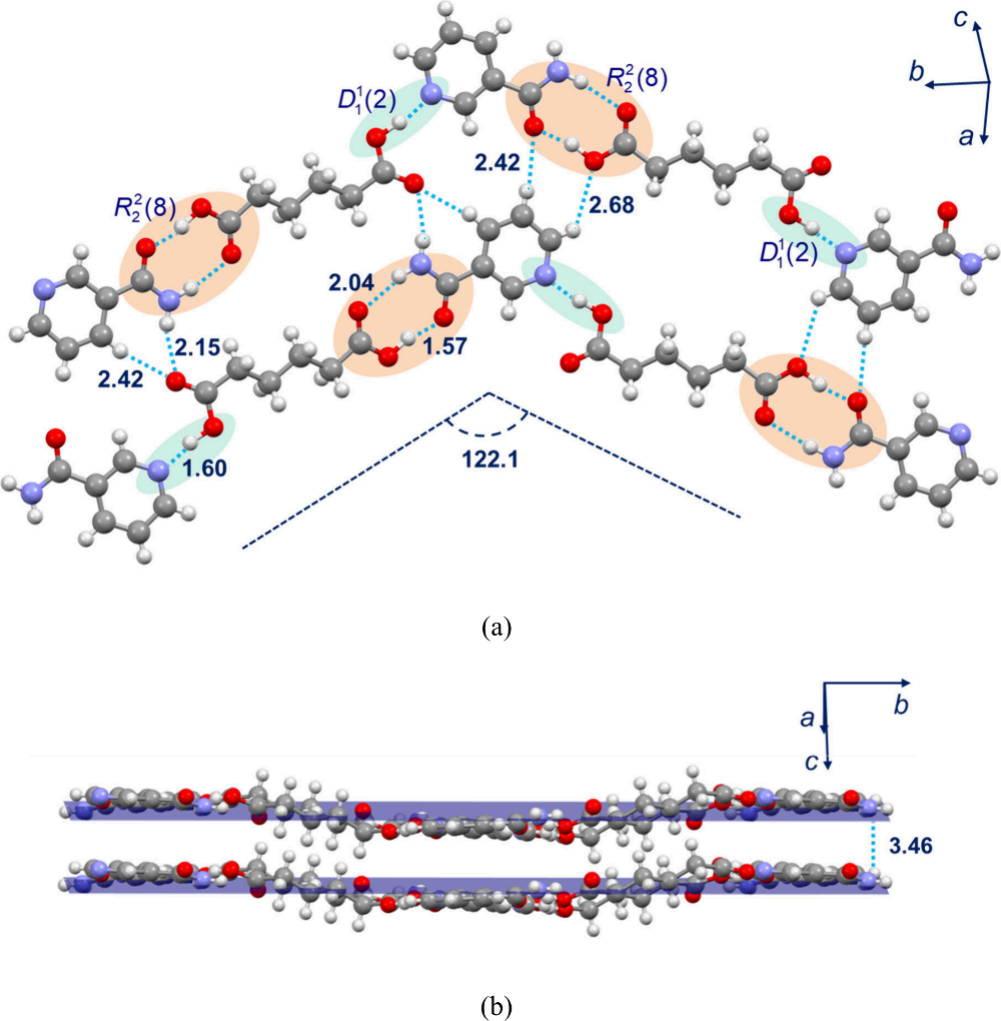
Crystalline structure of NIC:AA form II at 298 ± 2 K (distances in Å): (a) 1D chain sustained by *R*_2_^2^(8) and *D*_1_^1^(2) motifs and 2D sheets resulting from their interaction through nonclassic C–H···O and N–H···O hydrogen bonds; (b) separation between 2D planes containing the 1D chains in the crystal structure of form II of NIC:AA (distances in Å).

The 1D packing of both polymorphs (Figures 3a and 4a) exhibits infinite *zigzag* chains, where adipic acid and nicotinamide molecules alternate, defining two types of heterosynthons: *D*_1_^1^(2) established from an O–H···N hydrogen bond between the AA carboxylic group and the heterocyclic N of NIC; *R*_2_^2^(8) involving the COOH group of AA and the CONH_2_ group of NIC. The chain patterns are, however, different, namely:

$$\mathrm{Form I}:,\mathrm{NIC}\overset{D_{1}^{1}\left(2\right)}{\cdot \cdot \cdot }\mathrm{AA}\overset{D_{1}^{1}\left(2\right)}{\cdot \cdot \cdot }\mathrm{NIC}\overset{R_{2}^{2}\left(8\right)}{\cdot \cdot \cdot }\mathrm{AA}\overset{R_{2}^{2}\left(8\right)}{\cdot \cdot \cdot }\mathrm{NIC}$$

$$\mathrm{Form II}:,\mathrm{NIC}\overset{D_{1}^{1}\left(2\right)}{\cdot \cdot \cdot }\mathrm{AA}\overset{R_{2}^{2}\left(8\right)}{\cdot \cdot \cdot }\mathrm{NIC}\overset{D_{1}^{1}\left(2\right)}{\cdot \cdot \cdot }\mathrm{AA}\overset{R_{2}^{2}\left(8\right)}{\cdot \cdot \cdot }\mathrm{NIC}$$

In both forms the 1D chains are linked to each other via an equal number of nonclassical C–H···O hydrogen bonds, forming 2D sheets (Figures 3a and 4a). In form II, however, the sheet framework is further reinforced by a NH···O hydrogen bond (*d*_NH···O_ = 2.15 Å) between the amidic N–H group of one NIC molecule and the amidic C=O group of a NIC molecule in an adjacent chain (Figure 4a). In form I, the equivalent of the latter type of bond, strengthens the 3D packing which is completed by stacking layers of 2D sheets (Figures 3b and 4b).

On average, the H-bond distances sustaining the *R*_2_^2^(8) and *D*_1_^1^(2) motifs are slightly longer in form I (Figure 3a: *d*_OH···O_ = 1.63 Å, *d*_NH···O_ = 2.03 Å for *R*_2_^2^(8) and *d*_OH···N_ = 1.75 Å for *D*_1_^1^(2)) than in form II (Figure 4a: *d*_OH···O_ = 1.57 Å, *d*_NH···O_ = 2.04 Å for *R*_2_^2^(8) and *d*_OH···N_ = 1.60 Å for *D*_1_^1^(2)). The C–H···O hydrogen bond interactions sustaining the 2D sheets are also slightly longer, on average, in form I (*d*_CH···O_ = 2.60 Å, 2.53 Å) than in form II (*d*_CH···O_ = 2.42 Å, 2.68 Å). These two observations could suggest a higher lattice enthalpy, Δ_lat_ *H*_m_^o^, in form II than in form I, in contrast with the calorimetric evidence discussed below, which unequivocally indicated that the opposite is experimentally found. The fact that Δ_lat_ *H*_m_^o^ is larger in form I than in form II is, however, consistent with the following results, which indicate a more compact and strong 3D framework in the former than in the latter polymorph: (*i*) the distance between the planes of the 2D sheets is ∼ 2% shorter in form I (3.39 Å, Figure 3b) than in form II (3.46 Å, Figure 4b); (*ii*) the density and the packing index are both ∼ 3% larger in form I than in form II; and (*iii*) the intersheet interactions in form I are most likely strengthened by the presence of the NH···O hydrogen bond mentioned above, which in form II is engaged in 2D interactions.

### Thermal Analysis

The thermal behaviors of the two NIC:AA polymorphs and their precursors were investigated through a combination of differential scanning calorimetry (DSC), variable temperature powder X-ray diffraction (VT-PXRD), and solid-state nuclear magnetic resonance (ssNMR) experiments.

#### Differential Scanning Calorimetry

The DSC results obtained for the NIC:AA polymorphs and their precursors, in the range 298–435 K, at a heating rate of 5 K min^–1^, are illustrated in Figure 5 and summarized in Table 2 (detailed results are given as Supporting Information). The temperatures of solid–solid phase transition, *T*_trs_, or fusion, *T*_fus_, in Table 2, refer to the onsets of the observed peaks.

**Figure 5:**
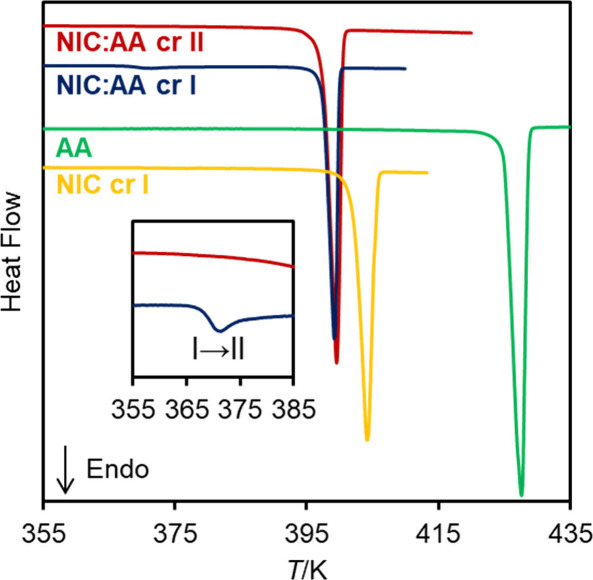
Typical DSC curves obtained for NIC:AA form II (red line), NIC:AA form I (blue line), AA (green line), and NIC form I (yellow line). The inset refers to the temperature range of the NIC:AA form I → form II phase transition.

**Table 2:** Temperatures, Standard Molar Enthalpies, and Standard Molar Entropies of Fusion and Phase Transition Obtained by DSC for NIC:AA Forms I and II and Their Precursors AA and NIC (*p*° = 0.1 MPa)^a^

	AA	NIC(crI)^b^	NIC:AA cr I	NIC:AA cr II
*T*_fus_/K	424.7 ± 0.5	401.8 ± 0.2	397.6 ± 0.2	397.8 ± 0.2
Δ_fus_ *H*_m_^o^/kJ mol^–1^	37.1 ± 0.2	23.6 ± 0.1	52.8 ± 0.1	52.2 ± 0.3
Δ_fus_ *S*_m_^o^/J K^–1^ mol^–1^	87.3 ± 0.6	58.8 ± 0.2	132.8 ± 0.3	131.3 ± 0.8
*T*_trs_ /K			367.2 ± 0.4^c^	
Δ_trs_ *H*_m_^o^/kJ mol^–1^			1.57 ± 0.34^c^	
Δ_trs_ *S*_m_^o^/J K^–1^ mol^–1^			4.29 ± 0.94^c^	

a The temperatures of solid–solid phase transitions (*T*_trs_) or fusion (*T*_fus_) refer to the onsets of the observed peaks.

b Revised data from reference^57^ (see Supporting Information).

c Form I → Form II phase transition.

In the case of the monoclinic NIC:AA form II, only a single peak, due to fusion, was detected, at *T*_fus_ = 397.8 ± 0.2 K, with Δ_fus_ *H*_m_^o^ = 52.2 ± 0.3 kJ mol^–1^ and Δ_fus_ *S*_m_^o^ = 131.3 ± 0.8 J K^–1^ mol^–1^. The triclinic form I showed, however, a different DSC pattern. A broad endothermic peak was first detected at *T*_trs_ = 367.2 ± 0.4 K, with Δ_trs_ *H*_m_^o^ = 1.57 ± 0.34 kJ mol^–1^ and Δ_trs_ *S*_m_^o^ = 4.29 ± 0.94 J K^–1^ mol^–1^. Variable-temperature powder X-ray diffraction and solid-state nuclear magnetic resonance analysis (see below) confirmed that this peak corresponded to the form I → form II phase transition, as previously reported.^29^ The phase transition peak was followed by a larger and sharper endothermic peak due to fusion of form II, as indicated by the close agreement between the obtained *T*_fus_ = 397.6 ± 0.2 K, Δ_fus_ *H*_m_^o^ = 52.8 ± 0.1 kJ mol^–1^, and Δ_fus_ *S*_m_^o^ = 132.8 ± 0.3 J K^–1^ mol^–1^, and the corresponding data mentioned above for the fusion of pure form II. The *T*_fus_ value obtained for NIC:AA form II (*T*_fus_ = 397.8 ± 0.2 K) is lower than the temperatures of fusion of both coformers, namely, *T*_fus_(AA) = 424.7 ± 0.5 K and *T*_fus_(NIC) = 401.9 ± 0.2 K. This observation places the NIC:AA system in the second most abundant group of cocrystals in terms of temperature of fusion (class II; 30–39% relative abundance), for which *T*_fus_(cocrystal) < *T*_fus_(precursors).^55,56^ It can also be noted that the DSC data here obtained for forms I and II agree with the cruder *T*_fus_ = 400 ± 5 K (*n* = 2, *n* is the number of data points considered), *T*_trs_ = 363 ± 6 K (*n* = 3), Δ_trs_ *H*_m_^o^ = 1.6 ± 0.2 kJ mol^–1^ (*n* = 3) calculated by combining published data^29^ determined in a single laboratory, at heating rates of 1 K min^–1^ and 5 K min^–1^, and two different instruments.

Overall, the DSC analysis suggests the two NIC:AA forms are enantiotropically related: polymorph I is thermodynamically stable up to *T*_trs_, where stability is reversed and polymorph II becomes the stable form up to fusion. The fact that the form II → form I transformation has been found to take months at ambient temperature and pressure,^29^ and it is not observed on cooling in the time scale of a typical DSC experiment, gives a clear indication that the forward and reverse form I ⇌ form II processes are hindered by a significant activation barrier.^58^ As a result the transition is only observed on heating (form I → form II), suggesting that the process requires form I to attain considerable metastability. This is consistent with the fact that the onset temperature observed in this work by DSC (*T*_trs_ = 367.2 ± 0.4 K) is 78 K higher than *T*_trs_ = 289.2 ± 0.7 K given by the solubility measurements discussed below, which should be representative of equilibrium conditions. This type of behavior is not uncommon,^59^ and has been observed, for example, in the case of 4’-hydroxybenzaldehyde and 4’-hydroxyacetophenone.^58,60^ Further support for the conclusions of the DSC study was obtained from the results of VT-PXRD and ssNMR experiments.

#### Variable Temperature Powder X-ray Diffraction (VT-PXRD)

VT-PRXD analyses evidenced a progressive transformation of form I to form II between 358 and 383 K (Figure 6), consistent with the crI → crII phase transition temperature found in the DSC experiments (*T*_trs_ = 367.2 ± 0.4 K). No intermediary phases were detected. Rietveld refinement of the diffraction data, performed with TOPAS,^33^ provided the temperature dependence of the specific volume, *v*, for both polymorphs over the 300–370 K range (see Supporting Information). The *v* (cm^3^ g^–1^) vs *T* (K) values were fitted by least-squares regression to the equation:

2 $$v=v_{o}\left(1+\alpha_{V}T\right)$$

where *α*_*V*_, is the thermal expansion coefficient and *v*_0_ is the specific volume at 0 K. The results and corresponding fitting relationships are illustrated in Figure 7, from which it can be concluded that *α*_*V*_ = (2.57 ± 0.08) × 10^–4^ K^–1^ and *v*_0_ = 0.685 ± 0.002 cm^3^ g^–1^ for form I and *α*_*V*_ = (2.81 ± 0.06) × 10^–4^ K^–1^ and *v*_0_ = 0.701 ± 0.002 cm^3^ g^–1^ for form II. These values indicate that both polymorphs exhibit positive thermal expansion, and that the corresponding *α*_*V*_ values are ca. 50–60% larger than the average value, *α*_*V*_ = (1.73 ± 0.45) × 10^–4^ K^–1^,^61^ found in a survey of thermal expansion coefficients for organic molecular crystals in the Cambridge Structural Database. Figure 7 also shows that form I has a smaller specific volume than form II at any given temperature. This reflects the larger crystal density and higher lattice enthalpy (see below) of form I compared with form II throughout the temperature range probed, suggesting that the phase transition is solely entropically driven, as generally expected for an enantiotropic polymorphic system.

**Figure 6:**
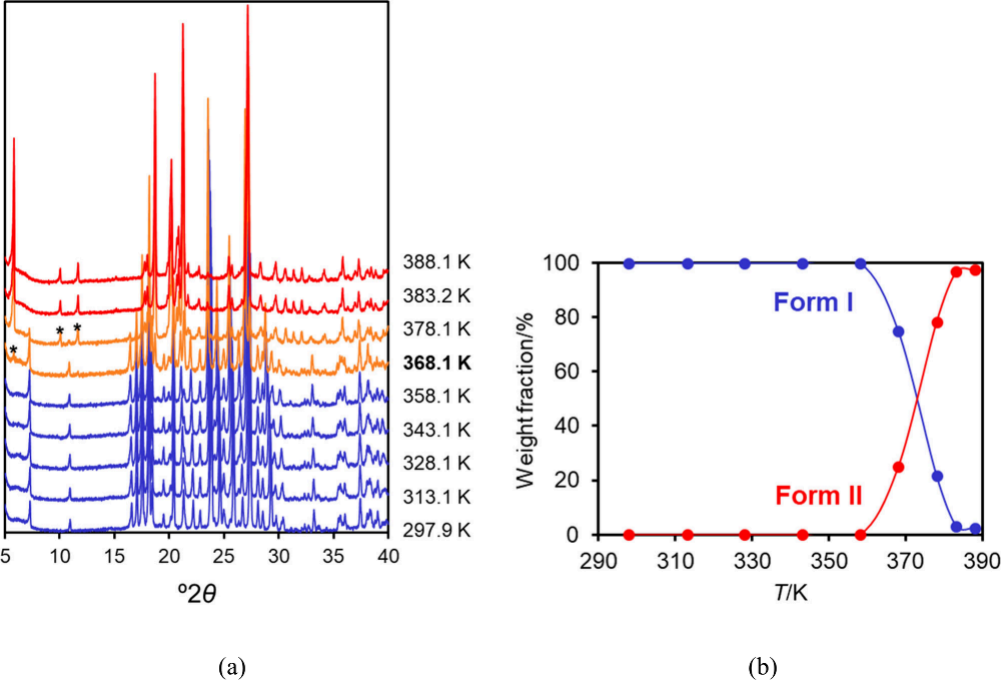
Variable-temperature powder X-ray diffraction results for NIC:AA. (a) Blue patterns refer to form I. Orange patterns refer to conditions where transition from form I to form II has started yet both forms are simultaneously present (* indicates characteristic peaks of form II). Red patterns refer to form II. (b) Quantitative phase analysis performed via Rietveld refinement of the VT-PXRD data, showing the form I → form II phase transition in the range 358–383 K.

**Figure 7:**
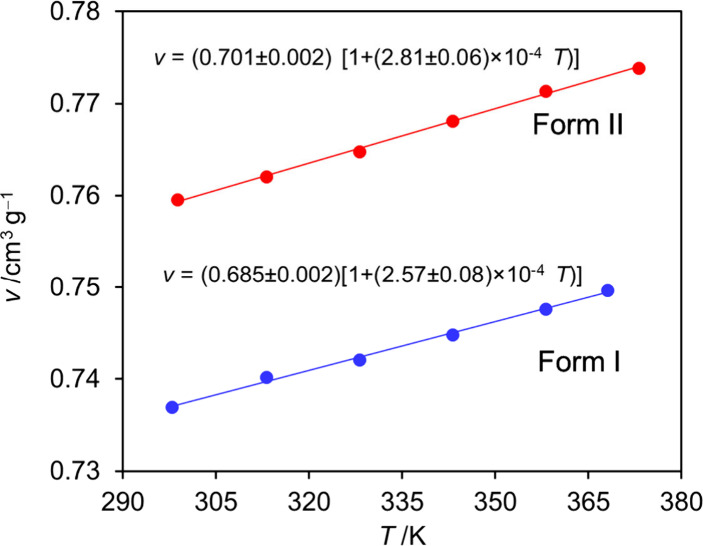
Specific volume of NIC:AA form I (blue) and form II (red) as a function of temperature.

#### Solid-State Nuclear Magnetic Resonance (ssNMR)

The results of the ssNMR experiments are shown in Figure 8. The comparison of the spectra of form I acquired near room temperature (313 K, Figure 8a) and at 363 K (Figure 8b) reveals significant spectral changes with temperature, consistent with the occurrence of the form I → form II phase transition. Cooling the sample to 313 K results in minimal spectral variation (Figure 8c), and the spectrum closely matches that of an independently prepared form II sample recorded at the same temperature (Figure 8d).

**Figure 8:**
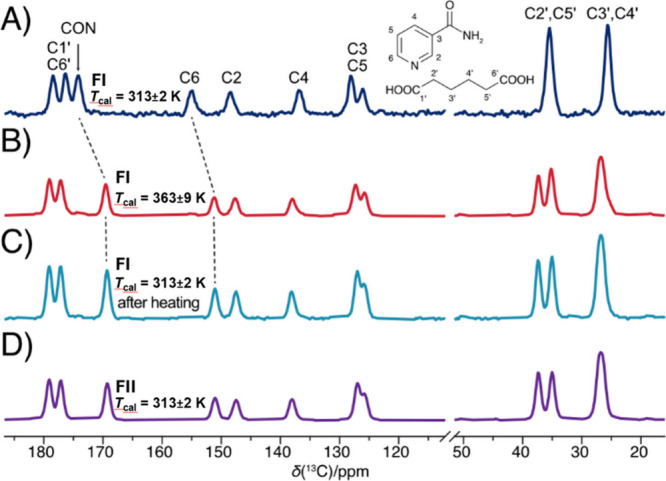
^13^C CP-MAS spectra of nicotinamide-adipic acid cocrystals (14.1 T, 18 kHz MAS, contact time 5 ms) for form I measured at temperatures of (a) 313 ± 2 K, (b) 363 ± 9 K, and (c) again at 313 ± 2 K after heating, and (d) for form II at 313 ± 2 K. The signal assignments are based on comparison with DFT-calculated chemical shifts.

The most significant chemical-shift differences between the two forms are observed for the carbonyl carbon of NIC, the C6 carbon of NIC, and the CH_2_ carbons C2′ and C5′ of AA (see atom labeling in the inset of Figure 8). In form I, the NIC carbonyl group participates in two classical hydrogen bonds: (NIC)­C=O–HO­(AA), 1.63 Å, in the 2D sheet plane (Figure 3a) and interplanar (NIC)­C=O–HN­(NIC), 2.19 Å (Figure 3b). In the case of form II, however, only one type of classical hydrogen bond interaction is formed (NIC)­C=O–HO­(AA), 1.57 Å (Figure 4). This difference is reflected in the deshielding of the carbonyl carbon in form I (i.e., a higher chemical shift, 174 ppm in form I, 169 ppm in form II; DFT calculated values: 172 ppm in form I, 166 ppm in form II). The C6 carbon of NIC is involved in a C–H···O intermolecular hydrogen bond in both forms; however, the shorter contact and larger C–H···O angle in form I result in reduced shielding of this carbon (i.e., 155 ppm in form I, 151 ppm in form II; DFT calculated values: 155 ppm in form I, 149 ppm in form II). Additionally, the two CH_2_ carbon atoms adjacent to the COOH groups in AA resonate at the same chemical shift in form I, whereas two distinct signals are observed in form II. A comparison of the most significant hydrogen bond distances and angles computed here by DFT and experimentally found by SCXRD is given in the Supporting Information.

In agreement with the VT-PRXD observations, no intermediate phases were detected during the solid–solid phase transitions. The ssNMR results also corroborate the conclusion from the DSC experiments, that due to a significant activation barrier, the form I → form II process is only observed on heating.

### Thermodynamic Stability of the NIC:AA Polymorphs

The relative stability of the two NIC:AA polymorphs and their stabilities relative to decomposition into the NIC­(crI) and AA coformers, at 298.15K, were evaluated based on the standard molar Gibbs energy, enthalpy, and entropy changes of the reaction:

3 NIC:AA(cr I/cr II)→NIC(cr)+AA(cr)

denoted as, Δ_r_ *G*_m_^o^(3), Δ_r_ *H*_m_^o^(3), and *T*Δ_r_ *S*_m_^o^(3), respectively. The values of Δ_r_ *H*_m_^o^(3) and Δ_r_ *G*_m_^o^(3) were obtained from solution calorimetry and solubility measurements, respectively, and the entropic contribution, *T*Δ_r_ *S*_m_^o^(3), was calculated from

4 $$\Delta_{r}G_{m}^{o}=\Delta_{r}H_{m}^{o}-T\Delta_{r}S_{m}^{o}$$

The solubility measurements also provided an estimate of the equilibrium temperature of the form I → form II phase transition.

The standard molar Gibbs energy of reaction 3 was evaluated from the equation:^20^

5 $$\Delta_{r}G_{m}^{o}\left(3\right)=-RT,\ln\left(\frac{x_{\mathrm{NIC}}x_{\mathrm{AA}}}{x_{\mathrm{NIC}}^{\prime }x_{\mathrm{AA}}^{\prime }}\right)$$

where *x*_NIC_ and *x*_AA_ are the molar fraction solubilities of NIC and AA in acetonitrile resulting from the dissolution of known amounts of NIC:AA­(crI/crII) samples, and *x*_NIC_^′^ and *x*_AA_^′^ are the corresponding solubilities of the pure coformers. The solubility measurements relied on the apparatus and procedure described in the experimental section. This dynamic technique was selected because once in contact with the solvent, both NIC:AA forms tend to convert to the more stable NIC_2_:AA phase.^29^ Consequently, equilibrium methods cannot be applied. To ensure that the NIC:AA­(crI/crII) samples were stable during the measurements, suspensions of the two polymorphs were stirred in several solvents for ca. 15 min (typical time of an experiment) and then analyzed by PXRD. The results showed that acetonitrile was the best solvent to avoid interference of the transformation into NIC_2_:AA (Figure S3 of the Supporting Information). For the sake of consistency *x*_NIC_^′^ and *x*_AA_^′^ were also determined by the same technique.

Linear least-squares fits of van’t Hoff equation:

6 $$\ln x=\frac{a}{T}+b$$

to the ln *x* vs 1/*T* data obtained for the two NIC:AA polymorphs led to the results illustrated in Figure 9.

**Figure 9:**
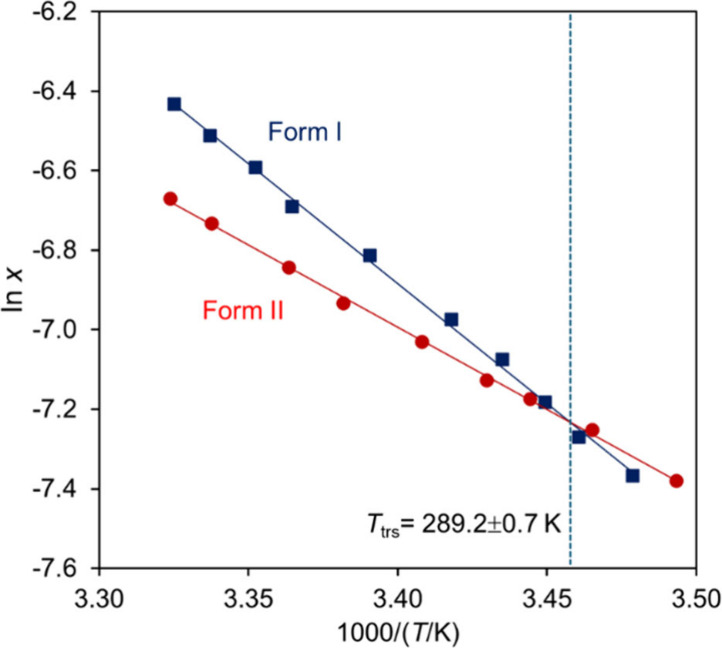
Molar fraction (*x*) solubilities of NIC:AA forms I (blue) and II (red) in acetonitrile, as a function of temperature (*T*). The vertical dashed line marks the intersection of the two curves, corresponding to the form I → form II phase transition temperature.

From those linear relationships and analogous counterparts for the coformers (*a* and *b* parameters are given in Table S18 of the Supporting Information), the molar fraction solubilities *x*_NIC_, *x*_AA_, *x*_′_^NIC^, and *x*_′_^AA^, at 298.15 K, in Table 3, were calculated.

**Table 3:** Results of the Solubility and Calorimetry Measurements on NIC:AA Polymorphs, and Their Co-Formers at 298.15 K

	NIC:AA(crI)	NIC:AA(crII)
*x*_NIC_×10^3^	1.35 ± 0.05	1.11 ± 0.02
*x*_AA_×10^3^	1.35 ± 0.05	1.11 ± 0.02
*x*_′_^NIC^×10^3^	7.94 ± 0.10	
*x*_′_^AA^×10^3^	1.79 ± 0.03	
Δ_r_ *G*_m_^o^(3)/kJ mol^–1^	5.09 ± 0.28	6.06 ± 0.16
Δ_r_ *H*_m_^o^(3)/kJ mol^–1^	6.47 ± 0.07	5.09 ± 0.10
*T*Δ_r_ *S*_m_^o^(3)/kJ mol^–1^	1.38 ± 0.29	–0.97 ± 0.19
*T*_trs_(crI → crII)	289.2 ± 0.7 K	
Δ_trs_ *G*_m_^o^(crI → crII)/kJ mol^–1^	–0.97 ± 0.33	
Δ_trs_ *H*_m_^o^(crI → crII)/kJ mol^–1^	1.38 ± 0.12	
*T*Δ_trs_ *S*_m_^o^(crI → crII)/kJ mol^–1^	2.35 ± 0.34	
Δ_lat_ *H*_m_^o^/kJ mol^–1^	247.8 ± 0.8	246.4 ± 0.8

To assess the accuracy of the dynamic solubility measurements the solubilities of pure NIC and AA were also determined under equilibrium conditions, at 296.05 ± 0.02 K, using the gravimetric method (see Supporting Information for details). The mean values obtained *x̅*_NIC_ = (7.89 ± 0.22) × 10^–3^ and *x̅*_AA_ = (1.76 ± 0.01) × 10^–3^ differ by 8.4% and 9.7%, respectively, from *x*_NIC_ = (7.23 ± 0.09) × 10^–3^ and *x*_AA_ = (1.59 ± 0.03) × 10^–3^ calculated at the same temperature, using eq 6 and the values of the *a* and *b* parameters for NIC and AA in Table S18 of the Supporting Information. Substitution of the *x*_NIC_, *x*_AA_, *x*_NIC_^′^, and *x*_AA_^′^ values in Table 3 into eq 5 led to the Δ_r_ *G*_m_^o^(3) values that are also given in Table 3, from which the Gibbs energy of the form I → form II phase transition at 298.15 K, could be derived as Δ_trs_ *G*_m_^o^(crI → crII) = Δ_r_ *G*_m_^o^(3, crI) – Δ_r_ *G*_m_^o^(3, crII) = –0.97 ± 0.33 kJ mol^–1^ (Table 3). Two main conclusions can be drawn from these results: (*i*) the fact that, at 298.15 K, Δ_r_ *G*_m_^o^(3) is always positive implies that, around ambient temperature and pressure, both NIC:AA polymorphs are stable relative to decomposition into the precursors; (*ii*) the negative value of Δ_trs_ *G*_m_^o^(crI → crII) indicates that the form I to form II transition is thermodynamically favored at that temperature. This is consistent with the prediction from the intersection of the solubility curves in Figure 9, that the crI → crII transition should occur at a temperature close to ambient conditions, namely *T*_trs_ = 289.2 ± 0.7 K (Table 3 and Figure 9). It is also worth recalling (see discussion of the DSC results) that the latter value, which corresponds to an estimate of the equilibrium temperature of the transition, is 78 K lower than the result obtained by DSC (*T*_trs_ = 367.2 ± 0.4 K, Table 2).

The standard molar enthalpy of reaction 3, Δ_r_ *H*_m_^o^(3), was obtained from isothermal calorimetry measurements of the enthalpies of the dissolution processes:

7 NIC:AA(crI/II)+2011DMSO(l)→[NIC+AA+2011DMSO](sln)

8 AA(cr)+2011DMSO(l)→[AA+2011DMSO](sln)

9 NIC(cr)+[AA+2011DMSO](sln)→[NIC+AA+2011DMSO](sln)

The corresponding results (see Supporting Information for details), Δ_sol_ *H*_m_^o^(7, crI) = 26.58 ± 0.04 kJ mol^–1^, Δ_sol_ *H*_m_^o^(7, crII) = 25.20 ± 0.08 kJ mol^–1^, Δ_sol_ *H*_m_^o^(8) = 9.41 ± 0.05 kJ mol^–1^, and Δ_sol_ *H*_m_^o^(9) = 10.70 ± 0.03 kJ mol^–1^ led to the Δ_sol_ *H*_m_^o^(3) values in Table 3, based on the equation:

10 $$\Delta_{r}H_{m}^{{}^{\circ}}\left(3,\mathrm{crI}/\mathrm{crII}\right)=\Delta_{\mathrm{sol}}H_{m}^{{}^{\circ}}\left(7,\mathrm{crI}/\mathrm{crII}\right)-\Delta_{\mathrm{sol}}H_{m}^{{}^{\circ}}\left(8\right)-\Delta_{\mathrm{sol}}H_{m}^{{}^{\circ}}\left(9\right)$$

From the Δ_r_ *H*_m_^o^(3) results it is also possible to obtain the enthalpy of the form I → form II phase transition as: Δ_trs_ *H*_m_^o^(crI → crII) = Δ_r_ *H*_m_^o^(3, crI) – Δ_r_ *H*_m_^o^(3, crII) = 1.38 ± 0.12 kJ mol^–1^. Finally, the entropy terms, *T*Δ_r_ *S*_m_^o^(3) and *T*Δ_trs_ *S*_m_^o^(crI → crII) could be derived from eq 4 by using the corresponding Gibbs energy and enthalpy data in Table 3. The fact that for both NIC:AA polymorphs Δ_r_ *H*_m_^o^(3) > |*T*Δ_r_ *S*_m_^o^(3)|, indicates that, as most commonly found,^11^ the larger stability of these cocrystals relative to the coformers is essentially of enthalpic origin, reflecting a lattice enthalpy gain when the cocrystals form as illustrated in Figure 10. The lattice enthalpies of NIC:AA­(crI/crII) are addressed in the following section.

**Figure 10:**
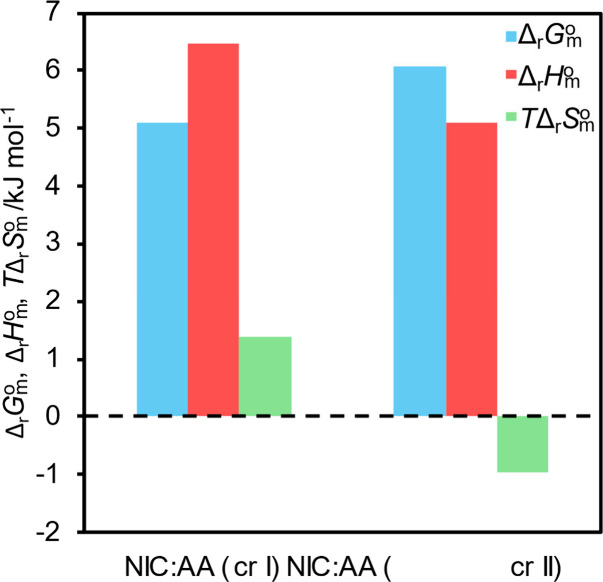
Comparison of the Gibbs energies, enthalpies, and entropies of reaction 3 (see also Table 3) for forms I and II of NIC:AA at 298.15 K.

### Lattice Enthalpies of the NIC:AA Polymorphs

The lattice enthalpies, Δ_lat_ *H*_m_^o^, of NIC:AA­(crI/crII), at 298.15 K, were calculated from

11 $$\Delta_{\mathrm{lat}}H_{m}^{o}\left(\mathrm{crI}/\mathrm{crII}\right)=\Delta_{r}H_{m}^{o}\left(3,\mathrm{crI}/\mathrm{crII}\right)+\Delta_{\mathrm{sub}}H_{m}^{o}\left(\mathrm{NIC}\right)+\Delta_{\mathrm{sub}}H_{m}^{o}\left(\mathrm{AA}\right)$$

which can be deduced from the thermodynamic cycle in Figure 11. By using the Δ_r_ *H*_m_^o^(3, crI/crII) values in Table 3, together with the previously reported enthalpy of sublimation of nicotinamide, Δ_sub_ *H*_m_^o^(NIC) = 108.0 ± 0.5 kJ mol^–1^,^57^ and the enthalpy of sublimation of adipic acid, Δ_sub_ *H*_m_^o^(AA) = 133.3 ± 0.6 mol^–1^, determined in this work (see Supporting Information) it was possible to obtain the lattice enthalpies of the two NIC:AA polymorphs summarized in Table 3. These values exceed the sum of the lattice enthalpies of pure NIC and AA (taken as their enthalpies of sublimation), by 6.5 kJ mol^–1^ for NIC:AA­(crI) and 5.1 kJ mol^–1^ for NIC:AA­(crII), reflecting the enthalpy gains associated with the formation of the cocrystals expressed by Δ_r_ *H*_m_^o^(3, crI/crII).

**Figure 11:**
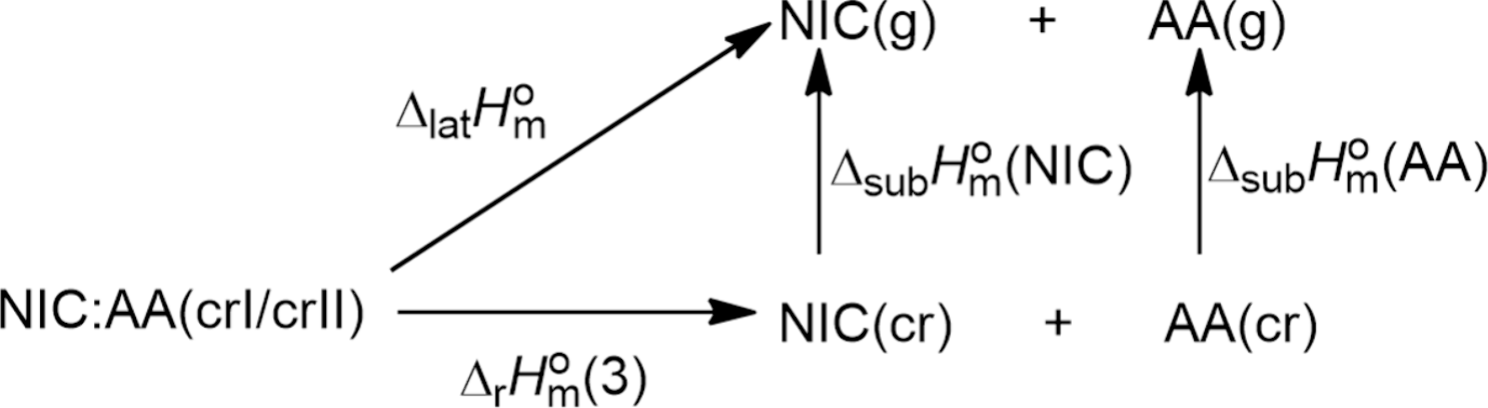
Thermodynamic cycle used to obtain the lattice enthalpies of the NIC:AA polymorphs.

## Conclusion

The polymorphism of the 1:1 nicotinamide–adipic acid cocrystal system (NIC:AA) was re-examined from structural and thermodynamic perspectives. Calorimetric and solubility measurements confirmed an enantiotropic relationship between forms I and II and enabled a quantitative assessment of their thermal stability domains, as well as their stabilities with respect to dissociation into the individual coformers. Overall, the thermodynamic analysis indicates that at 298 K: (*i*) both NIC:AA polymorphs are stable relative to dissociation into nicotinamide and adipic acid; (*ii*) stability is predominantly enthalpy-driven rather than entropy-driven, consistent with a net gain in lattice enthalpy upon cocrystal formation; and (*iii*) the form I → form II transformation is thermodynamically favored. The solubility studies further suggested that (*iv*) the equilibrium transition temperature is substantially lower (by ∼ 78 K) than the transition temperature inferred from DSC, solid-state NMR, or variable-temperature PXRD.

Single-crystal X-ray diffraction showed that at ∼ 298 K (*v*) form I has both a higher density (∼3%) and a higher packing index (∼3%) than form II, consistent with the relative lattice-enthalpy ordering, Δ_lat_ *H*_m_^o^(crI) > Δ_lat_ *H*_m_^o^(crII), observed at this temperature. Variable-temperature PXRD additionally revealed that both polymorphs exhibit thermal expansion coefficients that are approximately 50–60% higher than the average value reported from an analysis of Cambridge Structural Database data.

Finally, although cocrystallization is frequently pursued as a strategy to improve solubility and other physicochemical properties of organic functional materials, in the present case, the formation of NIC:AA does lead to a crystal form thermodynamically more stable than the individual coformers, but without any solubility benefit, at least when the solvent is acetonitrile. Indeed, depending on the NIC:AA polymorph the molar fraction solubilities of NIC and AA in this solvent were found to be lower by 83–86% and 26–38%, respectively, than for pure NIC and AA.

## Supplementary Material



## Notes

Tables S1–S4 with the indexation of the powder patterns corresponding to various samples mentioned in the text. Tables S5 and S6 with the VT-XRPD data. Table S7 and Figure S1 with SCXRD results. Figure S2 with a comparison of experimental and calculated ^13^C NMR spectra of forms I and II of nicotinamide-adipic acid cocrystals and magres files from the GIPAW calculations. Table S8 with a comparison of DFT computed hydrogen bond distances and angles and corresponding SCXRD results. Tables S9–S12 with detailed results of the DSC experiments. Tables S13–S16 with detailed results of the solution calorimetry experiments. Tables S17–S19 and Figure S3 with results related to the solubility experiments. Tables S20–S23 with the results of the enthalpy of sublimation measurements on adipic acid by Calvet microcalorimetry. Details of the assignment of uncertainties (Figure S4 and Tables S24 and S25). CIF files with single crystal X-ray diffraction structures of the two NIC:AA polymorphs at 298(2) K, deposited at the Cambridge Crystallographic Data Center (CCDC) with reference numbers 2535150 (form I) and 2535152 (form II) (PDF)
