# Supplementary material for: New Insights into the Structure and Thermodynamic Stability of Polymorphs I and II of the Nicotinamide:Adipic Acid Co-Crystal: A BEST-CSP Study
Source: Cryst Growth Des. 2026 Jun 23;26(14):5618–28. doi: 10.1021/acs.cgd.6c00576 (PMC13384025; doi:10.1021/acs.cgd.6c00576)
Supplement: Supplementary file 1 [file cg6c00576_si_001.pdf]

Supporting Information

New Insights into the Structure and Thermodynamic Stability of Polymorphs I and II of the Nicotinamide:Adipic acid Co-crystal: A BEST-CSP Study

Inês O. Feliciano,[a] Carlos E. S. Bernardes,[a] M. Fátima M. Piedade,[a] M. Soledade C. S. Santos,[a] Paolo P. Mazzeo,[b] Jan Blahut,[c] Martin Dračínský,[c] and Manuel E. Minas da Piedade[a,]*

[a] *Centro de Química Estrutural, Institute of Molecular Sciences, Departamento de Química e Bioquímica, Faculdade de Ciências, Universidade de Lisboa, 1749-016 Lisboa, Portugal.*

[b] *Department of Chemical Science, Live Science and Environmental Sustainability, University of Parma, Parco Area delle Scienze 17/A, 43124 Parma, Italy.*

[c] *Institute of Organic Chemistry and Biochemistry, Czech Academy of Science, Flemingovo nám. 2 160 00 Prague, Czech Republic.*

E-mail: memp@fc.ul.pt.

Table of Contents

		Page
1.	Powder X-Ray Diffraction	3
2.	Variable Temperature Powder X-ray Diffraction	5
3.	Single Crystal X-Ray Diffraction	6
4.	Solid-State Nuclear Magnetic Resonance	8
5.	Differential Scanning Calorimetry	9
6.	Solution calorimetry	12
7.	Solubility Measurements	14
8.	Enthalpy of Sublimation of Adipic Acid from Calvet Drop-Sublimation Microcalorimetry Experiments	18
9.	Assignment of Uncertainties	23
10.	References	29

1. Powder X-Ray Diffraction

Table S1. Indexation of the powder X-ray diffraction pattern of nicotinamide (form I) in the range 5º ≤ 2*θ* ≤ 35º (space group *P*2$_1$/*c*, *a* = 3.972±0.004 Å, *b* = 15.636±0.006 Å, *c* = 9.418±0.009 Å, *β* = 99.06±0.20º).

h	*k*	*l*	2*θ*(Obs)/º	Δ2*θ* /º
0	2	0	11.36	0.03
0	2	1	14.83	0.04
0	0	2	19.09	0.04
0	3	1	19.53	0.01
0	1	2	19.92	0.04
0	2	2	22.22	0.01
0	4	0	22.75	-0.01
1	1	0	23.35	0.00
0	4	1	24.71	0.01
1	2	0	25.39	0.01
0	3	2	25.62	-0.02
-1	2	1	25.85	0.01
1	1	1	26.59	0.00

h	*k*	*l*	2*θ*(Obs)/º	Δ2*θ* /º
-1	0	2	27.32	0.02
-1	1	2	27.91	0.01
1	3	0	28.47	0.01
-1	3	1	28.88	0.00
-1	2	2	29.64	0.00
0	5	1	30.16	0.00
1	3	1	31.19	-0.01
1	4	0	32.30	-0.01
1	1	2	32.54	0.00
0	3	3	33.54	-0.04
1	2	2	34.06	-0.01
-1	1	3	34.33	-0.03
0	6	0	34.43	0.00

Table S2. Indexation of the powder X-ray diffraction pattern of adipic acid (form I) in the range 5º ≤ 2*θ* ≤ 35º (space group *P*2$_1$/*n*, *a* = 7.364±0.010 Å, *b* = 5.178±0.011 Å, *c* = 10.029±0.011 Å, *β* = 110.66±0.40º).

h	*k*	*l*	2*θ*(Obs)/º	Δ2*θ* /º
-1	0	1	13.03	0.04
0	0	2	18.87	-0.05
1	1	0	21.44	-0.03
-1	1	1	21.54	-0.01
1	1	1	25.34	0.05
2	0	0	25.85	-0.02
-1	0	3	26.85	0.02
-2	1	1	29.74	-0.04
2	1	0	31.22	0.04

Table S3. Indexation of the powder X-ray diffraction pattern of NIC:AA (form I) in the range 5° ≤ 2θ ≤ 35° (space group $P\bar{1}$, a = 5.0516±0.0015 Å, b = 5.3999±0.0017 Å, c = 24.260±0.008 Å, α = 93.996±0.012°, β = 93.766±0.012°, γ = 90.933±0.012°).

h	*k*	*l*	2θ(Obs)/°	Δ2θ/°
0	0	2	7.34	0.01
0	0	3	11.01	0.01
0	0	4	14.62	-0.07
0	-1	1	16.62	0.00
0	1	1	17.11	0.00
-1	0	0	17.62	0.00
1	0	1	18.24	0.01
0	1	2	18.50	0.00
1	0	2	19.54	0.00
-1	0	3	20.19	0.00
0	1	3	20.46	-0.01
0	-1	4	21.34	-0.01
-1	0	4	22.26	0.01
0	1	4	22.89	0.00
1	-1	0	23.91	-0.01

h	*k*	*l*	2θ(Obs)/°	Δ2θ/°
-1	0	5	24.70	-0.01
-1	1	2	25.05	0.01
0	1	5	25.63	0.00
-1	-1	3	25.95	0.02
1	-1	3	26.41	0.01
0	-1	6	26.76	0.02
-1	0	6	27.45	-0.02
-1	1	4	28.16	-0.01
0	1	6	28.64	0.03
1	0	6	29.34	0.01
1	1	4	29.82	-0.03
-1	0	7	30.48	0.02
1	0	7	32.45	0.02
1	-1	6	32.80	-0.01
0	2	0	33.26	-0.01

Table S4. Indexation of the powder X-ray diffraction pattern of NIC:AA (form II) in the range 5° ≤ 2θ ≤ 35° (space group $P2_1/c$, a = 4.978±0.005 Å, b = 30.268±0.023 Å, c = 8.935±0.008 Å, β = 93.68±0.27°).

h	*k*	*l*	2θ(Obs)/°	Δ2θ/°
0	2	0	5.87	0.03
0	1	1	10.40	0.06
0	4	0	11.70	0.01
1	0	0	17.84	-0.01
1	1	0	18.11	0.02
1	2	0	18.80	0.01
0	1	2	20.09	-0.03
0	2	2	20.73	-0.02
1	1	1	21.21	-0.01
0	3	2	21.79	0.01
1	3	1	22.81	0.00

h	*k*	*l*	2θ(Obs)/°	Δ2θ/°
0	4	2	23.14	0.00
0	8	1	25.63	0.07
-1	0	2	25.95	-0.02
0	6	2	26.65	-0.02
1	1	2	27.88	0.01
-1	7	1	28.75	0.02
-1	5	2	29.96	0.04
1	5	2	31.49	0.02
1	9	0	32.06	-0.06
0	5	3	33.56	-0.01
1	10	0	34.64	-0.04

2. Variable Temperature Powder X-ray Diffraction (VT-PXRD)

Table S5. Unit cell parameters obtained from Rietveld refinement of the VT-XRPD data determined for NIC:AA form I.

T/K	*a*/Å	*b*/Å	*c*/Å	*α*/°	*β*/°	*γ*/°	*V*/Å^3
297.9	5.047(8)	5.397(7)	24.220(12)	93.819(6)	93.655(7)	91.161(3)	656.67(2)
313.1	5.051(3)	5.404(3)	24.257(10)	93.933(5)	93.769(5)	91.044(4)	659.46(3)
328.1	5.055(3)	5.411(8)	24.297(8)	94.066(4)	93.912(5)	90.909(8)	661.13(4)
343.1	5.058(6)	5.417(5)	24.350(7)	94.243(6)	94.100(6)	90.736(4)	663.57(3)
358.1	5.060(2)	5.424(6)	24.413(6)	94.442(2)	94.314(7)	90.562(3)	666.02(1)
368.1	5.063(6)	5.429(8)	24.456(9)	94.571(4)	94.444(7)	90.463(2)	667.94(5)

Table S6. Unit cell parameters obtained from Rietveld refinement of the VT-XRPD data obtained for NIC:AA form II.

T/K	*a*/Å	*b*/Å	*c*/Å	*β*/°	*V*/Å^3
298.8	4.989(3)	30.371(3)	8.953(2)	93.798(5)	1353.64(1)
313.1	4.992(5)	30.368(4)	8.976(2)	93.661(3)	1358.02(3)
328.1	4.993(5)	30.359(9)	9.007(4)	93.497(4)	1362.80(2)
343.1	4.993(7)	30.343(5)	9.050(5)	93.328(7)	1368.64(3)
358.1	4.991(3)	30.318(6)	9.098(5)	93.150(2)	1374.60(2)
373.1	4.989(5)	30.279(5)	9.141(6)	92.940(2)	1379.07(2)

3. Single Crystal X-Ray Diffraction

Table S7 Crystal data and structure refinement parameters for NIC:AA polymorphs

	NIC:AA(cr I)[a]	NIC:AA(cr II)[a]
T/K	298(2) K	298(2) K
λ/Å	0.71073	0.71073
Empirical formula	$C_{12}H_{16}N_2O_5$	$C_{12}H_{16}N_2O_5$
Formula weight	268.269	268.269
Crystal Color	Colorless	Colorless
Crystal system	Triclinic	Monoclinic
Space Group	$P\bar{1}$	$P2_1/c$
a/Å	5.0516(15)	4.981(5)
b/Å	5.3999(17)	30.35(3)
c/Å	24.260(8)	8.932(8)
α/deg	93.996(12)	90
β/deg	93.766(12)	93.76(3)
γ/deg	90.933(12)	90
V/Å^3	658.59(36)	1347(2)
Z/Z'	2/1	4/1
ρ_{calc}/g cm^{-3}	1.35345(7)	1.323
k^a	0.721	0.694
μ/mm^{-1}	0.106	0.104
F(000)	284	568
θ limits/deg	2.530 to 26.817	2.382 to 25.433
Limiting indices	$-6 \le h \le 6$	$-5 \le h \le 6$
	$-6 \le k \le 6$	$-36 \le k \le 35$
	$-30 \le l \le 30$	$-9 \le l \le 10$
No. of refns collected/unique	16730 / 2797 [R(int) = 0.0485]	20525 / 2471 [R(int) = 0.0972]
Completeness to θ	99.4%	100.0%
Data / restraints / parameters	297 / 0 / 236	2471 / 0 / 189
GOF on F^2	1.095	0.961
Final R indices	R_1 = 0.0494	R_1 = 0.0540
[$l > 2\sigma(l)$]	wR_2 = 0.1300	wR_2 = 0.1136
R indices	R_1 = 0.0681	R_1 = 0.1397
(all data)	wR_2 = 0.1381	wR_2 = 0.1322
Extinction coefficient	–	0.0077(18)
Largest diff peak and hole/e Å^3	0.242 and −0.151	0.196 and -0.159

[a]Value of Kitaigorodskii's packing index, k, was calculated using Mercury 2025.1.1.[1]

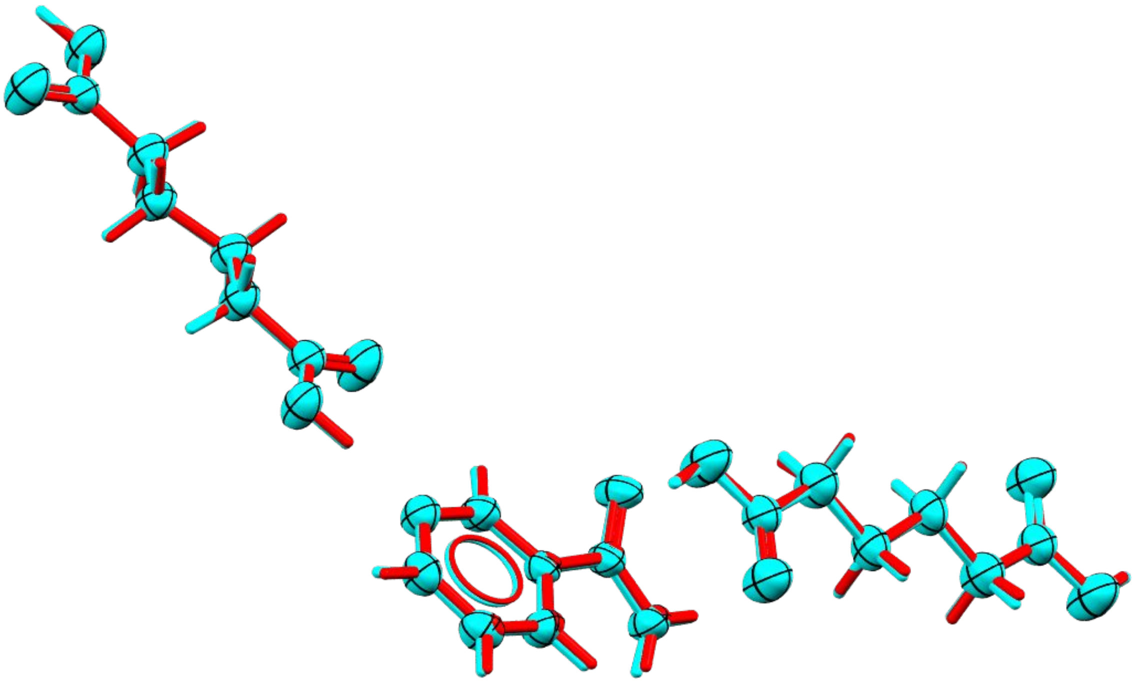

Figure S1. Overlay of supramolecular structure of forms I of NIC:AA at 299(2) K (red, this work) and 180 K (blue, reference 2).

4. Solid-State Nuclear Magnetic Resonance (ssNMR)

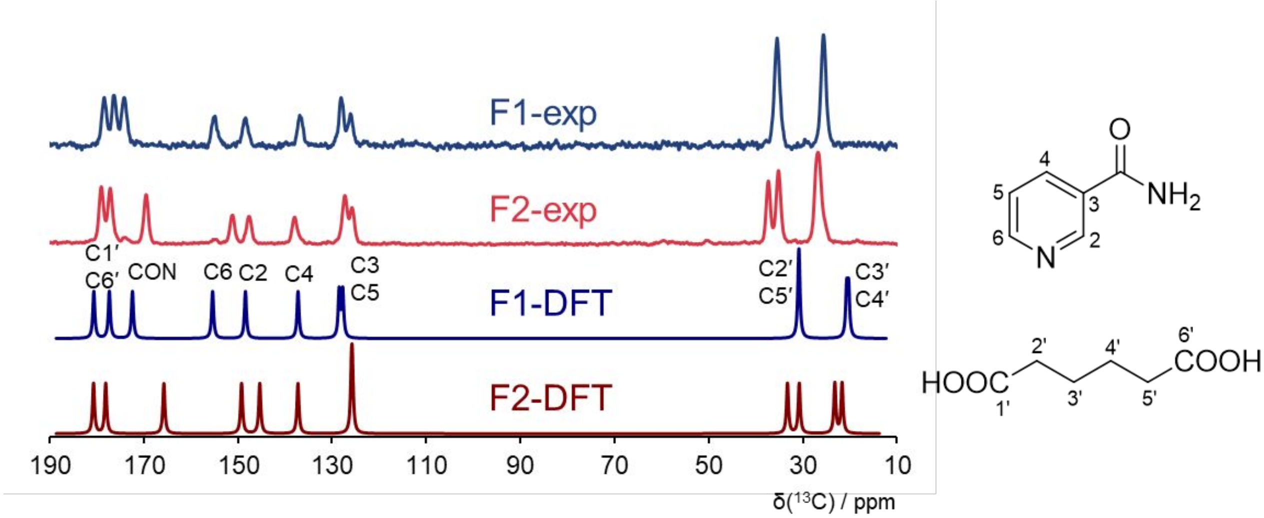

Figure S2. Experimental and calculated ^{13}C NMR spectra of form I and form II of nicotinamide-adipic acid co-crystals.

Table S8 Comparison of DFT computed and SCXRD hydrogen bond distances and angles

	Distance/Å		Angle/°	
Bond type	Experimental	Computed	Experimental	Computed
			**Form I**	
OH···O	1.63	1.58	166.9	167.5
NH···O	2.03	1.89	168.5	168.4
OH···N	1.75	1.63	172.4	176.0
			**Form II**	
OH···O	1.57	1.55	167.5	167.4
NH···O	2.09	1.91	168.9	169.7
OH···N	1.60	1.52	168.9	172.4

5. Differential Scanning Calorimetry

The results of the DSC experiments on NIC:AA forms I and II and adipic acid (AA) are summarized in Tables S9-S11. For comparison purposes previously reported data for nicotinamide (form I) are also listed in Table S12, which further includes unpublished entropy data. In those tables m and M refer to the mass and molar mass of the sample, respectively; T_{fus} and T_{trs} denote the peak onset temperatures of a fusion or solid-solid phase transition, respectively, and T_{max} is the temperature of the peak maximum; $\Delta_{fus}h^o$, $\Delta_{fus}H^o_m$, and $\Delta_{fus}S^o_m$, $\Delta_{trs}h^o$, $\Delta_{trs}H^o_m$, and $\Delta_{trs}S^o_m$ are the corresponding standard specific enthalpies, standard molar enthalpies and standard molar entropies of fusion and solid-solid phase transition, respectively. The uncertainties indicated for the mean values $\overline{\Delta_{fus}h^o}$ and $\overline{\Delta_{trs}h^o}$ are standard errors of the mean (u), and those assigned to $\bar{T}_{fus}$, $\bar{T}_{trs}$, $\bar{T}_{max}$, $\overline{\Delta_{fus}H^o_m}$, $\overline{\Delta_{fus}S^o_m}$, $\overline{\Delta_{trs}H^o_m}$ and $\overline{\Delta_{trs}S^o_m}$ represent expanded uncertainties calculated as described in Section 9.

Table S9. Results of the DSC Experiments on NIC:AA form I (p^o = 1 bar)

m/g	T_{trs}/K	T_{max}/K	$\Delta_{trs}h$/ J g^{-1}	$\Delta_{trs}H^o_m$ / kJ mol^{-1}	$\Delta_{trs}S^o_m$/ J K^{-1} mol^{-1}	T_{fus}/K	T_{max}/K	$\Delta_{fus}h$/J g^{-1}	$\Delta_{fus}H^o_m$/ kJ mol^{-1}	$\Delta_{fus}S^o_m$/ J K^{-1} mol^{-1}
2.306	367.0	370.4	6.58	1.77	4.82	397.8	399.6	197.12	52.88	132.93
2.504	366.8	370.3	7.50	2.01	5.48	397.5	399.2	196.99	52.85	132.96
3.474	366.9	370.3	6.51	1.75	4.77	397.8	400.1	196.23	52.64	132.33
2.37	367.4	370.3	4.62	1.24	3.38	397.4	399.3	197.16	52.89	133.09
2.950	368.0	371.1	4.11	1.10	2.99	397.7	399.3	196.60	52.74	132.61

M = 268.269 g mol^{-1}

$\bar{T}_{trs} \pm U$ = 367.2±0.4 K

$\bar{T}_{max} \pm 2u$ = 370.5±0.3 K

$\overline{\Delta_{trs}h^o} \pm U$ = 5.86±0.64 J g^{-1}

$\overline{\Delta_{trs}H^o_m} \pm U$ = 1.57±0.34 kJ mol^{-1}

$\overline{\Delta_{trs}S^o_m} \pm U$ = 4.29±0.94 J K^{-1} mol^{-1}

$\bar{T}_{fus} \pm U$ = 397.6±0.2 K

$\bar{T}_{max} \pm U$ = 399.5±0.3 K

$\overline{\Delta_{fus}h^o} \pm u$ = 196.82±0.18 J g^{-1}

$\overline{\Delta_{fus}H^o_m} \pm U$ = 52.8±0.1 kJ mol^{-1}

$\overline{\Delta_{fus}S^o_m} \pm U$ = 132.8±0.3 J K^{-1} mol^{-1}

Table S10. Results of the DSC Experiments on NIC:AA form II (p^o = 1 bar)

m/g	T_{fus}/K	T_{max}/K	$\Delta_{fus}h$/J g^{-1}	$\Delta_{fus}H^o_m$/kJ mol^{-1}	$\Delta_{fus}S^o_m$/J K^{-1} mol^{-1}
2.081	397.9	399.6	193.14	51.85	130.32
2.529	397.4	399.5	193.23	51.88	130.54
3.023	398.0	399.9	195.83	52.57	132.10
1.741	397.7	399.5	196.00	52.62	132.30
2.707	398.1	400.3	195.17	52.40	131.63

M = 268.269 g mol^{-1}

$\bar{T}_{fus} \pm u$ = 397.8±0.2K

$\bar{T}_{max} \pm u$ = 399.8±0.3 K

$\overline{\Delta_{fus}h^o} \pm u$ = 194.67±1.24 J g^{-1}

$\overline{\Delta_{fus}H^o_m} \pm U$ = 52.2±0.3 kJ mol^{-1}

$\overline{\Delta_{fus}S^o_m} \pm U$ = 131.3±0.8 J K^{-1} mol^{-1}

Table S11. Results of the DSC Experiments on Adipic Acid form I (p^o = 1 bar)

m/g	T_{fus}/K	T_{max}/K	$\Delta_{fus}h$/J g^{-1}	$\Delta_{fus}H^o_m$/kJ mol^{-1}	$\Delta_{fus}S^o_m$/J K^{-1} mol^{-1}
3.469	425.0	427.7	254.24	37.16	87.44
5.499	425.2	428.7	253.79	37.09	87.23
4.457	424.2	427.1	256.52	37.49	88.38
4.351	424.0	426.9	252.49	36.90	87.03
4.848	425.0	427.6	251.88	36.81	86.61

M = 146.141 g mol^{-1}

$\bar{T}_{fus} \pm U$ = 424.7±0.5 K

$\bar{T}_{max} \pm U$ = 427.6±0.6 K

$\overline{\Delta_{fus}h^o} \pm u$ = 253.78±0.81 J g^{-1}

$\overline{\Delta_{fus}H^o_m} \pm U$ = 37.1±0.2 kJ mol^{-1}

$\overline{\Delta_{fus}S^o_m} \pm U$ = 87.3±0.6 J K^{-1} mol^{-1}

Table S12. DSC results for Nicotinamide form I (p^o = 1 bar)[a]

m/g	T_{fus}/K	T_{max}/K	$\Delta_{fus}h$/J g^{-1}	$\Delta_{fus}H^o_m$/kJ mol^{-1}	$\Delta_{fus}S^o_m$/J K^{-1} mol^{-1}
4.186	401.6	403.6	193.97	23.69	58.99
4.705	402.0	404.5	193.75	23.66	58.86
5.440	402.0	404.4	192.56	23.52	58.51
1.141	401.7	403.1	194.79	23.79	59.22
2.158	402.0	403.6	194.15	23.71	58.98
1.922	402.2	403.8	193.75	23.66	58.83
3.562	401.9	404.1	192.90	23.56	58.62
2.985	401.2	403.8	191.28	23.36	58.23

[a] Data from reference 3.

M = 122.127 g mol^{-1}

$\bar{T}_{fus} \pm U$ = 401.8±0.2 K

$\bar{T}_{max} \pm U$ = 403.9±0.4 K

$\overline{\Delta_{fus}h^o} \pm u$ = 193.39±0.39 J g^{-1}

$\overline{\Delta_{fus}H^o_m} \pm U$ = 23.6±0.1 kJ mol^{-1}

$\overline{\Delta_{fus}S^o_m} \pm U$ = 58.8±0.2 J K^{-1} mol^{-1}

6. Solution calorimetry

The results of the solution calorimetry studies of the processes:

NIC:AA(cr I/II) + 2011DMSO(l) → [NIC + AA + 2011DMSO](sln) (S1)
AA (cr) + 2011DMSO (l) → [AA + 2011DMSO](sln) (S2)
NIC (cr) + [AA + 2011DMSO](sln) → [NIC + AA + 2011DMSO](sln) (S3)

are listed in Tables S13 to S16. In those tables, m, m_{DMSO}, and m_{sln} are the masses of compound, DMSO, and [AA + 2011DMSO] solution, respectively; A is the area of the calorimetric curve; $A_{\mathrm{b}} = -1.294 \pm 0.019$ mV s is the area corresponding to the effect of opening of the drop-chamber; ε is the energy equivalent of the calorimeter; the value $\bar{n}$ = 2011 is the number of DMSO molecules per mole of adipic acid in solution; and $\Delta_{\mathrm{sol}}h^{\mathrm{o}}$ is the standard specific enthalpy of solution at 298.15 K.

Table S13. Solution calorimetry results for the dissolution of NIC:AA form I in DMSO at 298.15 K (eq. S1).[a]

m/mg	m_{DMSO}/g	$(A - A_{\mathrm{b}})$/(mV·s)	$\Delta_{\mathrm{sol}}h^{\mathrm{o}}$ /J g^{-1}
24.2392	14.1433	333.997	99.114
24.1926	14.1754	333.501	99.157
24.1843	14.1702	332.545	98.907
24.2643	14.2249	334.759	99.237
24.2294	14.2124	333.542	99.019

[a] $\bar{\varepsilon}$ = 7.193±0.003 μW mV^{-1}; $\bar{n}$ = 2011±2; $\overline{\Delta_{\mathrm{sol}}h^{\mathrm{o}}}$ = 99.087±0.057 J g^{-1}; M(NIC:AA)= 268.269 g mol^{-1}; $\Delta_{\mathrm{sol}}H^{\mathrm{o}}_{\mathrm{m}}$ = 26.58±0.04 kJ mol^{-1}

Table S14. Solution calorimetry results for the dissolution of NIC:AA form II in DMSO at 298.15 K (eq. S1).[a]

m/mg	m_{DMSO}/g	$(A - A_{\mathrm{b}})$/(mV·s)	$\Delta_{\mathrm{sol}}h^{\mathrm{o}}$ /J g^{-1}
24.2612	14.1967	317.109	93.756
24.2457	14.2133	318.240	94.150
24.1629	14.1135	315.106	93.542
24.1416	14.1630	316.035	93.901
24.2387	14.2127	318.695	94.312

[a] $\bar{\varepsilon}$ = 7.173±0.002 μW mV^{-1}; $\bar{n}$ = 2011±2; $\overline{\Delta_{\mathrm{sol}}h^{\mathrm{o}}}$ = 93.932±0.137 J g^{-1}; M(NIC:AA)= 268.269 g mol^{-1}; $\Delta_{\mathrm{sol}}H^{\mathrm{o}}_{\mathrm{m}}$ = 25.20±0.08 kJ mol^{-1}

Table S15. Solution calorimetry results for the dissolution of AA form I in DMSO at 298.15 K (eq. S2).[a]

m/mg	m_{DMSO} /g	$(A - A_b)$/(mV·s)	$\Delta_{sol}h^o$ /J g^{-1}
13.1120	14.13017	117.659	64.375
13.1102	14.22378	117.656	64.382
13.2968	14.16083	119.562	64.507
13.1835	14.17181	118.606	64.541
13.2390	14.19573	118.240	64.072

[a] $\bar{\varepsilon}$ = 7.174±0.017 μW mV^{-1}; $\bar{n}$ = 2011±6; $\overline{\Delta_{sol}h^o}$ = 64.375±0.083 J g^{-1}; M(AA)= 146.142 g mol^{-1}; $\Delta_{sol}H^o_m$ = 9.41±0.05 kJ mol^{-1}

Table S16. Solution calorimetry results for the dissolution of NIC form I in a (AA + 2011 DMSO) solution at 298.15 K (eq. S3).[a]

m/mg	m_{sln} /g	$(A - A_b)$/(mV·s)	$\Delta_{sol}h^o$ /J g^{-1}
10.9806	14.14328	133.924	87.522
10.9648	14.23689	133.268	87.218
11.0346	14.17413	135.257	87.960
11.0708	14.18499	135.329	87.719
11.0357	14.20897	134.489	87.452

[a] $\bar{\varepsilon}$ = 7.176±0.004 μW mV^{-1}; $\bar{n}$ = 2011±6; $\overline{\Delta_{sol}h^o}$ = 87.574±0.125 J g^{-1}; M(NIC)= 122.127 g mol^{-1}; $\Delta_{sol}H^o_m$ = 10.70±0.03 kJ mol^{-1}

7. Solubility Measurements

Dynamic Method. The results of the molar fraction, x, solubility measurements on forms I and II of NIC:AA and on the pure co-formers NIC and AA, carried out with the apparatus illustrated in Figure 1 of the main text, in acetonitrile, and corresponding to the processes:

$$\mathrm{NIC:AA(cr\ I)}\ f\quad \mathrm{NIC(sln)} + \mathrm{AA(sln)} \tag{S4}$$

$$\mathrm{NIC:AA(cr\ II)}\ f\quad \mathrm{NIC(sln)} + \mathrm{AA(sln)} \tag{S5}$$

$$\mathrm{NIC(cr)}\ f\quad \mathrm{NIC(sln)} \tag{S6}$$

$$\mathrm{AA(cr)}\ f\quad \mathrm{AA(sln)} \tag{S7}$$

are given in Table S17. These results were fitted to the van't Hoff equation (T in K):

$$\ln x = \frac{a}{T} + b \tag{S8}$$

leading to the a and b parameters in Table S18.

The stability of the NIC:AA polymorphs during the time required for the measurements (~15 minutes) was assessed using slurry tests. In these experiments form I or form II suspensions in acetonitrile were kept under stirring for 15 minutes, at 298 K. Then the suspended solids were filtered and analyzed by PXRD. As shown in Figure S3 the analyses indicated that no significant conversion of both forms to the more stable NIC_2:AA phase[4] occurred within the timeframe typical of the solubility measurements.

Table S17. Molar fraction solubilities of nicotinamide and adipic acid in acetonitrile, in equilibrium with the NIC:AA polymorphs and with the pure co-formers

NIC:AA, form I		NIC:AA, form II	
T/K	$(x_{NIC} = x_{AA})\times10^4$	T/K	$(x_{NIC} = x_{AA})\times10^4$
287.47	6.312	286.27	6.233
288.97	6.959	288.61	7.083
289.91	7.596	290.34	7.668
291.13	8.457	291.57	8.031
292.58	9.341	293.43	8.839
294.92	10.988	295.71	9.742
297.22	12.410	297.32	10.663
298.30	13.686	299.62	11.903
299.66	14.823	300.86	12.679
300.74	16.044		
**NIC, form I**		**AA, form I**	
T/K	$x'_{NIC}\times10^4$	T/K	$x'_{AA}\times10^4$
290.74	57.326	290.42	11.651
292.31	61.174	292.11	12.500
293.14	63.136	292.99	13.581
294.30	66.879	294.65	14.684
294.72	67.972	295.30	15.230
295.93	71.153	296.53	16.175
296.59	73.751	297.48	17.118
297.80	78.134	297.81	17.481
298.63	80.662	298.96	18.914
299.30	83.245	299.86	19.677
299.93	86.747	301.84	21.812
300.56	88.737	302.55	22.529
301.44	91.647	304.04	24.528
303.68	100.951	305.01	25.887

Table S18. Parameters of equation S8 and corresponding coefficients of determination (R^2) for the different studied solubility processes.

Process	x	$-a$	b	R^2
Eq S4	x_{NIC}(crI) = x_{AA}(cr I)	6024.16±94.64	13.597±0.322	0.998
Eq S5	x_{NIC}(crII) = x_{AA}(cr II)	4137.78±57.74	7.074±0.183	0.999
Eq S6	x'_{NIC}	3922.21±39.18	8.319±0.132	0.999
Eq S7	x'_{AA}	4882.73±49.03	10.049±0.165	0.999

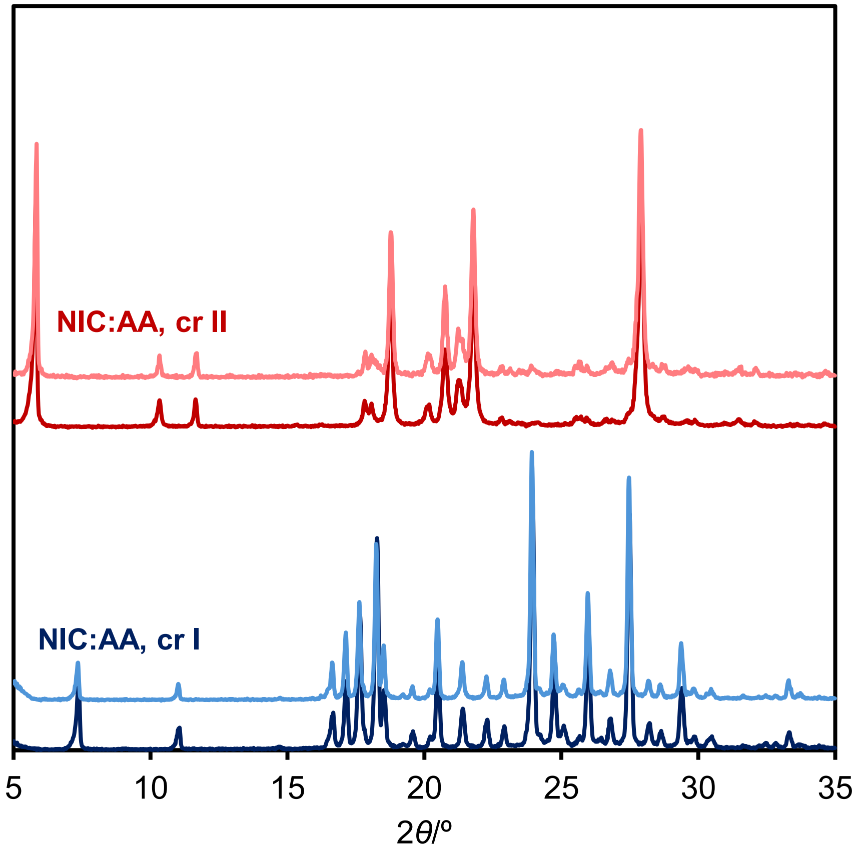

Figure S3. Powder X-ray diffraction patterns of NIC:AA form I (in blue) and form II (red), before (dark) and after 15 minutes (light) stirring under suspension in acetonitrile.

Solubility of pure NIC and AA in Acetonitrile. The accuracy of the dynamic method was assessed by measuring the solubilities of pure NIC and AA in acetonitrile, at 296.05±0.02 K, under equilibrium conditions, by the gravimetric method. Saturated solutions of NIC and AA in CH_3CN were prepared in 20 mL glass flasks at room temperature. The flasks were then transferred to a temperature-controlled system consisting of a MonoAluBlock™ holder adapted to a VELP AREX-6 Digital PRO hot plate, and kept at 296.05±0.02 K, under magnetic stirring, for one week. At the end of the equilibration period stirring was stopped and a sample of the saturated solution (1 cm^3) was withdrawn using a syringe equipped with a LabFil syringe filter (25 mm PTFE hydrophobic membrane, 0.22 μm pore size). This aliquot was transferred to a previously weighed glass vial of 1 cm^3 volume, which was weighed a second time when loaded with the solution and

a third time after the solution was taken to dryness. The weightings were performed with a precision of ±0.01 mg on a Mettler Toledo XS205 balance. The mole fraction, x, of NIC or AA in the saturated solutions was computed from:

$$x = \frac{M_{\mathrm{CH_3CN}}(m_3 - m_1)}{M_{\mathrm{CH_3CN}}(m_3 - m_1) + M(m_2 - m_3)} \tag{S9}$$

where m_1 is the mass of the empty vial, m_2 is the mass of the vial containing the extracted sample of saturated solution, m_3 is the mass of the vial plus the solid residue, and M and $M_{\mathrm{CH_3CN}}$ represent the molar masses of NIC (122.127 g.mol^{-1}) or AA (146.142 g mol^{-1}) and of the acetonitrile (41.053 g mol^{-1}) solvent, respectively. The measurements were performed in triplicate. The obtained results and corresponding mean values, with uncertainties given as twice the mean deviation, are summarized in Table S19. The mean value $\bar{x}_{\mathrm{NIC}}$ = (7.89±0.22)×10^{-3} is in good agreement with x_{NIC} = (8.54±1.16)×10^{-3} at 296.05 K, calculated from a fit of van't Hoff equation to previously reported x_{NIC} vs T data in acetonitrile.[5] A larger discrepancy is found , however, between $\bar{x}_{\mathrm{AA}}$ = (1.76±0.01)×10^{-3} here obtained and x_{AA} = (3.01±0.71)×10^{-3} at 296.05 K, also calculated from a fit of van't Hoff equation to published x_{AA} vs T data in acetonitrile.[6]

Table S19. Equilibrium molar fraction solubilities of pure nicotinamide form I and adipic acid form I in acetonitrile at 296.05±0.02 K, obtained by the gravimetric method

$x_{\mathrm{NIC}}\times10^3$	$x_{\mathrm{AA}}\times10^3$
8.061	1.753
7.720	1.756
7.890	1.761
$\bar{x}_{\mathrm{NIC}}\times10^3$ = 7.89±0.22	$\bar{x}_{\mathrm{AA}}\times10^3$ = 1.76±0.01

8. Enthalpy of Sublimation of Adipic Acid from Calvet Drop-Sublimation Microcalorimetry Experiments

The standard molar enthalpy of sublimation, $\Delta_{\text{sub}}H_{\text{m}}^{\text{o}}$, of adipic acid form I was determined by Calvet drop-sublimation microcalorimetry. The apparatus and general procedure have been described.[7, 8] The CBCAL 3.0[9] and EasyGraph II[10] programs were used for instrument control/data acquisition and data analysis, respectively. In a typical experiment, a mass of 2.2-3.5 mg of sample was placed into a glass capillary (Aldrich, Kimble melting point capillary, O.D.×L 1.5-1.8 mm×10-15 mm) and weighed with a precision of 0.1 μg in a Mettler XP2U balance. The capillary was equilibrated for ~15 min at the initial temperature T_{i} = 298.19±0.03 K inside the drop furnace placed above the entrance of the calorimetric cell, while the initial baseline was being recorded. The capillary was subsequently dropped into the calorimetric cell whose temperature was set to T_{f} = 374.96±0.01 K. When the signal returned to the baseline, the sample and reference cells were simultaneously evacuated to 1.3 Pa and the curve associated with the sublimation process was acquired. The corresponding standard specific enthalpy of sublimation, $\Delta_{\text{sub}}h^{\text{o}}$, which refers to the isothermal process:

$$\text{AA(crI, } T_{\text{f}}) \rightarrow \text{AA(g, } T_{\text{f}}) \tag{S10}$$

was calculated from:

$$\Delta_{\text{sub}}h^{\text{o}} = \frac{\varepsilon(A - A_{\text{b}})}{m} \tag{S11}$$

where m is the mass of the sample, A is the area of the sublimation curve, A_{b} is the area of the blank contribution, determined in separate experiments where the system was evacuated without sample inside, and ε is the calibration constant of the calorimeter. The value of ε was obtained from a series of electrical calibrations as described in the solution calorimetry section. Examination of the capillaries and calorimetric cell at the end of the experiments revealed no residual material indicative of partial unsublimed sample or sample decomposition.

The results in Table S20 led to $\Delta_{\text{sub}}H_{\text{m}}^{\text{o}}(374.96\text{ K}) = 130.20\pm0.21$ kJ mol^{-1}. Correction of this value to 298.15 K yielded $\Delta_{\text{sub}}H_{\text{m}}^{\text{o}}(298.15\text{ K}) = 133.3\pm0.6$ kJ mol^{-1}. The correction was based on the equation:

$$\Delta_{\text{sub}}H_{\text{m}}^{\text{o}}(298.15\text{ K}) = \Delta_{\text{sub}}H_{\text{m}}^{\text{o}}(374.96\text{ K}) + \Delta_{\text{g, 374.96 K}}^{\text{g, 298.15 K}}H_{\text{m}}^{\text{o}} - \Delta_{\text{cr, 374.96 K}}^{\text{cr, 298.15 K}}H_{\text{m}}^{\text{o}} \quad \text{(S12)}$$

where $\Delta_{\text{cr, 374.96 K}}^{\text{cr, 298.15 K}}H_{\text{m}}^{\text{o}}$ and $\Delta_{\text{g, 374.96 K}}^{\text{g, 298.15 K}}H_{\text{m}}^{\text{o}}$ are the enthalpy contributions associated with the cooling the crystalline and gaseous phases from 374.96 K to 298.15 K. The term $\Delta_{\text{g, 374.96 K}}^{\text{g, 298.15 K}}H_{\text{m}}^{\text{o}} = -14.19\pm0.28$ kJ mol^{-1} was obtained from:

$$\Delta_{\text{g, 374.96 K}}^{\text{g, 298.15 K}}H_{\text{m}}^{\text{o}} = \int_{374.96\text{ K}}^{298.15\text{ K}} C_{p,\text{m}}^{\text{o}}(\text{g})\text{d}T \quad \text{(S13)}$$

where $C_{p,\text{m}}^{\text{o}}(\text{g})$ represents the standard molar heat capacity of gaseous AA given by:

$$C_{p,\text{m}}^{\text{o}}(\text{g}) = 46.361 + 0.31381T + 5.0950\times10^{-4}T^2 - 6.5104\times10^{-7}T^3 \quad \text{(S14)}$$

with $C_{p,\text{m}}^{\text{o}}(\text{g})$ in J K^{-1} mol^{-1} and the temperature T in K. Equation S14was found by fitting $C_{p,\text{m}}^{\text{o}}(\text{g})$ values calculated in the temperature range 280–480 K, using statistical mechanics[11] and vibration frequencies obtained at the B3LYP/aug-cc-pVTZ level of theory,[12-15] scaled by 0.968.[16]

The $\Delta_{\text{cr, 374.96 K}}^{\text{cr, 298.15 K}}H_{\text{m}}^{\text{o}}$ term was experimentally obtained as $\Delta_{\text{cr, 374.96 K}}^{\text{cr, 298.15 K}}H_{\text{m}}^{\text{o}} = -17.32\pm0.48$ kJ mol^{-1} from the Calvet microcalorimetry experiments (Table S21), based on the area, A_{heat}, of the peak corresponding to heating the glass capillary containing the sample, using the expression:

$$\Delta_{\text{cr, 374.96 K}}^{\text{cr, 298.15 K}}H_{\text{m}}^{\text{o}} = \frac{M}{m}[\varepsilon A_{\text{heat}} - m_{\text{glass}}c_{p,\text{glass}}^{\text{o}}(T_{\text{f}} - T_{\text{i}})] \quad \text{(S15)}$$

Here m and M are the mass and molar mas of sample, respectively, m_{glass} and $c^{\text{o}}_{p,\text{glass}}$ are the mass and specific heat capacity of the glass capillary, respectively, ε is the calibration constant, and T_{i} = 298.19±0.03 and T_{f} = 374.96±0.01 K are the initial and final temperatures of the heating process, respectively.

The value of $c^{\text{o}}_{p,\text{glass}}$ (Table S22) was obtained from:

$$c^{\text{o}}_{p,\text{glass}}\Delta^{\text{cr, 298.15 K}}_{\text{cr, 374.96 K}}H^{\text{o}}_{\text{m}} = \frac{\varepsilon A_{\text{glass}}}{m_{\text{glass}}(T_{\text{f}} - T_{\text{i}})} \quad \text{(S16)}$$

based on the results of independent experiments, where an empty capillary of known mass was dropped into the calorimeter, under similar conditions to those present in the heating period of the main experiment, and an area A_{glass} was observed.

The previously reported standard molar enthalpies of sublimation of adipic acid at 298.15 K (Table S23) span a range of 119-155 kJ mol^{-1}, with most values showing an uncertainty larger than 5 kJ mol^{-1}.[17-22] The value obtained in this work lies between the values reported in the literature. The reason for these discrepancies was impossible to assess from the published information.

Table S20. Results of the Enthalpy of Sublimation Measurements on Adipic Acid form I by Calvet Microcalorimetry (p^o = 1 bar).

m/mg	m_{cap} /mg	A-A_b/mV s	T_i /K	T_f /K	$\Delta_{sub}h^o$ /J g^{-1}
2.6178	22.0512	149.290	298.28	374.98	891.702
3.2210	25.0638	183.111	298.15	374.96	888.893
2.7788	22.4074	158.228	298.10	374.94	890.331
2.2698	25.1550	129.253	298.13	374.95	890.387
2.7160	27.6977	155.145	298.19	374.96	893.169
2.7531	26.5630	156.866	298.28	374.95	890.907

M = 146.142 g mol^{-1}

$\overline{T}_i \pm u$ = 298.20±0.03 K

$\overline{T}_f \pm u$ = 374.96±0.01 K

$\overline{\varepsilon} \pm u$ = 15.636±0.007 W V^{-1}

$\overline{A_b} \pm u$ = −(4.565±0.071) mV s (5)

$\overline{\Delta_{sub}h^o} \pm u$ = 890.90±0.59 J g^{-1} (overall uncertainty u_c = 0.71 J g^{-1})

$\overline{\Delta_{sub}H^o_m} \pm U_c$ = 130.20±0.21 kJ mol^{-1}

Table S21. Enthalpy change, $\Delta^{cr, 298.15 K}_{cr, 374.96 K}H^o_m$, associated with heating the sample between the temperatures of the drop furnace, T_i, and of the calorimetric cell T_f (p^o = 1 bar).

m/mg	m_{cap} /mg	A_{aq} /mV s	T_i /K	T_f /K	$-\Delta^{cr, 298.15 K}_{cr, 374.15 K}h^o_m$ /J g^{-1}
2.6178	22.0512	107.491	298.28	374.98	124.523
3.2210	25.0638	120.938	298.15	374.96	108.333
2.7788	22.4074	108.268	298.10	374.94	112.901
2.2698	25.1550	115.398	298.13	374.95	113.008
2.7160	27.6977	130.676	298.19	374.96	125.199
2.7531	26.5630	126.689	298.28	374.95	126.986

$\overline{T}_i \pm u$ = 298.20±0.03 K

$\overline{T}_f \pm u$ = 374.96±0.01 K

$\Delta^{cr, 298.15 K}_{cr, 374.96 K}h^o \pm u$ = −118.49±3.26 J g^{-1}

$\Delta^{cr, 298.15 K}_{cr, 374.96 K}H^o_m \pm U$ = −17.32±0.48 kJ mol^{-1}

Table S22. Standard specific heat capacity values of the glass capillaries $c_p^{\mathrm{o}}(\mathrm{glass})$ (p^{o} = 1 bar).

m_{cap} /mg	T_{i} /K	T_{f} /K	A_{glass} / mV s	$c_p^{\mathrm{o}}(\mathrm{glass})$ /J K^{-1} g^{-1}
22.4048	298.24	374.98	88.063	0.801
25.0604	298.24	374.96	98.371	0.800
26.5662	298.23	374.95	103.987	0.798
25.1515	298.14	374.96	98.995	0.801
22.0382	298.22	374.98	87.087	0.805

$\overline{T}_{\mathrm{i}} \pm u$ = 298.21±0.02 K

$\overline{T}_{\mathrm{f}} \pm u$ = 374.97±0.01 K

$\overline{c_{p,\mathrm{glass}}^{\mathrm{o}}} \pm u$ = 0.801±0.001 J g^{-1} K^{-1}

Table S23. Enthalpies of sublimation of adipic acid obtained in this work and reported in the literature.

T/K^a	$\Delta_{\mathrm{sub}}H_{\mathrm{m}}^{\mathrm{o}}(T)$ / kJ mol^{-1}	$-\Delta_{\mathrm{cr},T}^{\mathrm{cr},\,298.15\,\mathrm{K}}H_{\mathrm{m}}^{\mathrm{o}}$ /kJ mol^{-1} ^{b}	$-\Delta_{\mathrm{g},T}^{\mathrm{g},\,298.15\,\mathrm{K}}H_{\mathrm{m}}^{\mathrm{o}}$ /kJ mol^{-1}	$\Delta_{\mathrm{sub}}H_{\mathrm{m}}^{\mathrm{o}}(298.15\ \mathrm{K})$ /kJ mol^{-1} ^{c}	Reference
374.96	130.20±0.21	17.32±0.48	14.19±0.19	133.3±0.6	This work
382.7	129.36±1.26	19.06±0.12	15.75±0.32	132.7±1.3	[17]
298				122.01±0.01	[18]
305.0	154±6	1.55±0.01	1.16±0.02	154.8±6.0	[19]
328	145±5	11.25±0.07	8.92±0.18	147.8±5.0	[20]
298				119±26	[21]
363.0	124.7±20.0	14.63±0.09	11.82±0.24	127.5±20.0	[22]

^{a} Reference temperature of the experimental measurements. ^{b} Correction of literature values to 298.15 K, based on the average heat capacity value in the temperature range 298.20±0.03 K to 374.96±0.01 K, $C_{p,\mathrm{m}}^{\mathrm{o}}(\mathrm{cr})$ = 225.6±6.3 J mol^{-1} K^{-1}, calculated from the data in Table S21.

9. Assignment of Uncertainties

Unless otherwise stated, all uncertainties of thermodynamic data were calculated as recommended in NIST Technical Note 1297.[23]

Powder and Single Crystal X-ray diffraction. The indicated uncertainties for the lattice parameters correspond to standard deviations (u), calculated as described in SHELXL-97.[24]

Differential Scanning Calorimetry. The uncertainties indicated for the mean values $\overline{\Delta_{\text{fus}}h^{\text{o}}}$ and $\overline{\Delta_{\text{trs}}h^{\text{o}}}$ in Tables S9 to S112 are standard errors of the mean (u_y) calculated from:[23]

$$u_y = \sqrt{\frac{\sum_{i=1}^{n}(y_i - \overline{y})^2}{n(n-1)}} \qquad \text{(S17)}$$

where y_i refers to the result of each individual experiment and $\overline{y}$ is the mean value of the n determinations. Those assigned to $\overline{T}_{\text{fus}}$, $\overline{T}_{\text{trs}}$, $\overline{T}_{\text{max}}$, $\overline{\Delta_{\text{fus}}H_{\text{m}}^{\text{o}}}$, $\overline{\Delta_{\text{fus}}S_{\text{m}}^{\text{o}}}$, $\overline{\Delta_{\text{trs}}H_{\text{m}}^{\text{o}}}$ and $\overline{\Delta_{\text{trs}}S_{\text{m}}^{\text{o}}}$ represent expanded uncertainties calculated as twice the standard errors of the mean ($U_y = 2u_y$).

Solution Calorimetry. The uncertainties assigned to the mean values of the standard specific enthalpies of solution, $\overline{\Delta_{\text{sol}}h^{\text{o}}}$, in Tables S13-S16 are combined standard uncertainties, u_{c}, calculated as:[23]

$$u_{\text{c}} = \overline{\Delta_{\text{sol}}h^{\text{o}}}\sqrt{\left(\frac{u_{\overline{\Delta_{\text{sol}}h^{\text{o}}}}}{\overline{\Delta_{\text{sol}}h^{\text{o}}}}\right)^2 + \left(\frac{u_{\overline{\varepsilon}}}{\overline{\varepsilon}}\right)^2} \qquad \text{(S18)}$$

where $u_{\overline{\varepsilon}}$ and $u_{\overline{\Delta_{\text{sol}}h^{\text{o}}}}$ are the uncertainties of the calibration and the main experiments, respectively, given through eq S17. The uncertainties of the corresponding standard molar enthalpies of solution, $\Delta_{\text{sol}}H_{\text{m}}^{\text{o}} = M\overline{\Delta_{\text{sol}}h^{\text{o}}}$, where M is the molar mass of the dissolved compound calculated

from the conventional standard atomic masses recommended by IUPAC in 2021,[25] were computed as expanded uncertainties, U, given by:[23]

$$U = 2Mu_\mathrm{c} \tag{S19}$$

Calvet-Drop Microcalorimetry. The uncertainties indicated for the mean values of $\overline{T_\mathrm{i}}$, $\overline{T_\mathrm{f}}$, $\overline{\varepsilon}$, $\overline{\Delta_{\mathrm{cr,\ 374.15\ K}}^{\mathrm{cr,\ 298.15\ K}} h^\mathrm{o}}$, and $\overline{c_p^\mathrm{o}(\mathrm{glass})}$ in Tables S20-S22 are standard errors of the mean (u_y) calculated from eq S17. That quoted for the molar quantity $\overline{\Delta_{\mathrm{cr,\ 374.15\ K}}^{\mathrm{cr,\ 298.15\ K}} H_\mathrm{m}^\mathrm{o}}$ is also a standard error of the mean obtained as:

$$u_{\overline{\Delta_{\mathrm{cr,\ 374.15\ K}}^{\mathrm{cr,\ 298.15\ K}} H_\mathrm{m}^\mathrm{o}}} = Mu_{\overline{\Delta_{\mathrm{cr,\ 374.15\ K}}^{\mathrm{cr,\ 298.15\ K}} h^\mathrm{o}}} \tag{S20}$$

where M = 146.142 g mol^{-1} is the molar mass of adipic acid calculated from the conventional standard atomic masses recommended by IUPAC in 2021,[25] The uncertainty assigned to $\overline{\Delta_\mathrm{sub} h^\mathrm{o}}$ corresponds to a combined standard uncertainty, u_c, given by:[23]

$$u_\mathrm{c} = \overline{\Delta_\mathrm{sub} h^\mathrm{o}} \sqrt{\left(\frac{u_{\overline{\Delta_\mathrm{sub} h^\mathrm{o}}}}{\overline{\Delta_\mathrm{sub} h^\mathrm{o}}}\right)^2 + \left(\frac{u_{\overline{\varepsilon}}}{\overline{\overline{\varepsilon}}}\right)^2} \tag{S21}$$

where $u_{\overline{\varepsilon}}$ and $u_{\overline{\Delta_\mathrm{sub} h^\mathrm{o}}}$ are the uncertainties of the calibration and the main experiments, respectively, calculated through eq S17. That of the standard molar enthalpy of sublimation represents an expanded uncertainty calculated through eq S19.

Solubility Measurements. The uncertainty assigned to the mole fraction solubilities of NIC and AA determined under equilibrium conditions by the solid residue method (Table S19) are mean deviations calculated from:

$$u_y = \frac{\sum_{i=1}^{n}(y_i - \bar{y})}{n} \tag{S22}$$

where y_i refers to the result of each individual experiment and $\bar{y}$ is the mean value of the n determinations (in this case $n = 3$, since the experiments were carried out in triplicate).

The uncertainty assigned to the temperature of the Form I $\rightarrow$ Form II transition, obtained from the intersection of the linear regressions corresponding to the temperature dependencies of the solubilities the two NIC:AA polymorphs determined by the dynamic method (Figure 9 of the main text) and given by van't Hoff equation were calculated as follows.[26]

If the intersecting lines corresponding to the NIC:AA forms I and II are given by:

$$y = a_1x + b_1 \tag{S23a}$$

$$y = a_2x + b_2 \tag{S23b}$$

where $y = \ln x$ (with x representing the molar fraction solubility) and $x = 1/T$, then, the intersection point, x_{int} is given by:

$$x_{\text{int}} = \frac{b_2 - b_1}{a_1 - a_2} \tag{S24}$$

Since in this case (Table S18) a_1 = 6024.54, b_1 =13.597 and a_2 = 4137.93, b_2 =7.0741, eq S24 yields $x_{\text{int}} = 1/T_{\text{trs}}$ = 0.003458 K^{-1}, which implies T_{trs} = 289.2 K.

The confidence interval of a particular x_{p} value, calculated from its y_{p} counterpart using one of the regression lines in eqs S23a or S23b is given by:

$$u_{x_{\text{p}}} = \pm t\frac{s_{y/x}}{a}\sqrt{\frac{1}{n} + \frac{1}{m} + \frac{(y_{\text{p}} - \bar{y})^2}{a^2\sum_i (x_{\text{i}} - \bar{x})^2}} \tag{S25}$$

$$s_{y/x} = \sqrt{\frac{\sum_i (y_i - \hat{y})^2}{n-1}} \tag{S26}$$

where *a* is the slope of the line; *n* represents the number of data points; *m* is the number of replicates for each data point (in this case *m* = 1); *t* is the Student's factor for 95% probability and *n* – 2 degrees of freedom; $\bar{x}$ and $\bar{y}$ are the mean values of the $x_i = 1/T_i$ and $y_i = \ln x_i$ coordinates of the experimental data points; and $(y_i - \hat{y})$ is the difference between an experimental y_i value and the corresponding value calculated from the regression line.

The lines representing the confidence intervals of eqs S23a and S23b, are given by:

$$y = (a_1 x + b_1) \pm t \frac{s_{(y/x)_1}}{a_1} \sqrt{\frac{1}{n_1} + 1 + \frac{(y_p - \bar{y}_1)^2}{a_1^2 \sum_i (x_i - \bar{x}_1)^2}} \tag{S27a}$$

$$y = (a_2 x + b_2) \pm t \frac{s_{(y/x)_2}}{a_2} \sqrt{\frac{1}{n_2} + 1 + \frac{(y_p - \bar{y}_2)^2}{a_2^2 \sum_i (x_i - \bar{x}_2)^2}} \tag{S27b}$$

Eqs S27a and S27b, together with the solubility data in Table S17 and the parameters of the corresponding van't Hoff equations in Table S18, lead to the results shown in Figure S4. Details of the calculations are summarized in Tables S24 and S25. In this case, the confidence limit lines are, to a good approximation, described by linear relationships. The corresponding fitting parameters and intersection *x* coordinates are:

Form I (+): $a_{1+} = -6023.63$; $b_{1+} = 13.5558$ (R^2 =0.999985)   Form II (+): $a_{2+} = -4138.27$; $b_{2+} = 7.0522$ (R^2 =0.999984)

Form I (–): $a_{1-} = -6025.27$; $b_{1-} = 13.6398$ (R^2 =0.999985)   Form II (–): $a_{2-} = -4137.46$; $b_{2-} = 7.0955$ (R^2 =0.999984)

Upper limit: $x_{int+} = 0.0034495$ K^{-1}; $T_{int} = 289.9$ K
Lower limit: $x_{int-} = 0.0034666$ K^{-1}; $T_{int} = 288.5$ K

It can therefore be concluded that the temperature of the form I → form II phase transition and associated uncertainty corresponding expressed as an expanded uncertainty is:

$$T_{trs} \pm U = 289.2 \pm 0.2 \text{ K}$$

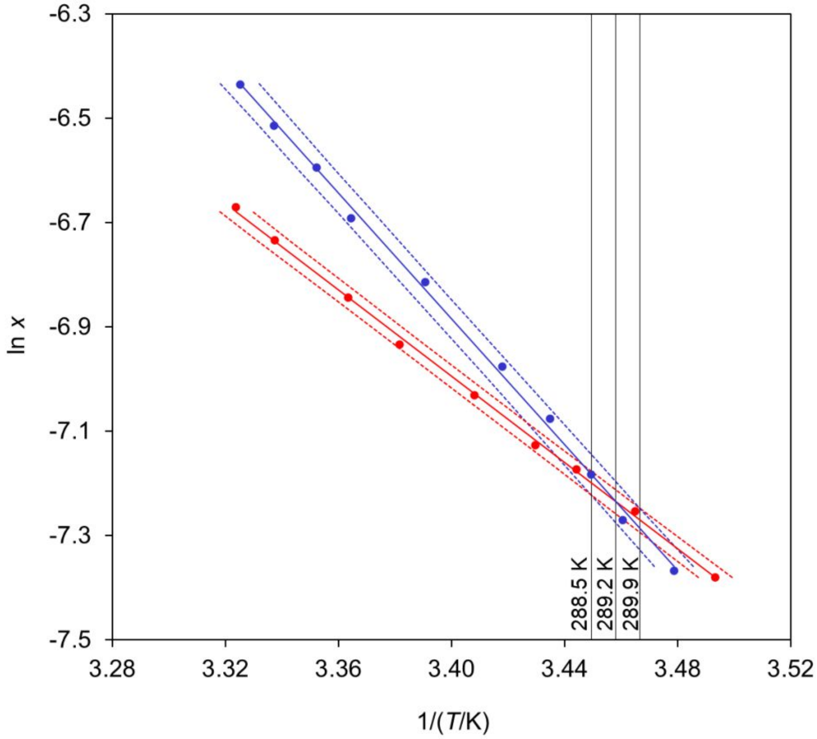

Figure S4. Confidence limits of the regression lines resulting from fitting van't Hoff equation to the solubility data for NIC:AA forms I and II in Table S16.

Table S24

	Form I	Form II
a	–6024.24	–4137.93
n	10	9
m	1	1
$\overline{y}$	–6.892069099	–7.016222847
$\overline{x}$	0.003401	0.00341
$\sum_i (x_i - \overline{x}_2)^2$	2.69866812×10^{-8}	2.69411372×10^{-8}
$\sum_i (y_i - \hat{y})^2$	1.92853761×10^{-3}	5.44218353×10^{-4}
$s_{y/x}$	0.015526339	0.00881734
t	2.306	2.365

Table S25

T/K	x_p×10[3]	tu_{xp}×10[6]	(x_p+tu_{xp})×10[3]	(x_p–tu_{xp})×10[3]	y_p
			Form I		
287.47	3.478623856	–6.834760856	3.471789096	3.485458617	–7.359038568
288.97	3.460566841	–6.593720640	3.453973120	3.467160561	–7.250253355
289.91	3.449346349	–6.472693729	3.442873655	3.455819043	–7.182655053
291.13	3.434891629	–6.351713888	3.428539915	3.441243343	–7.095572016
292.58	3.417868617	–6.262459580	3.411606158	3.424131077	–6.993016199
294.92	3.390750034	–6.244316458	3.384505717	3.396994350	–6.829639209
297.22	3.364511137	–6.372146632	3.358138990	3.370883283	–6.671561922
298.3	3.352329869	–6.478076659	3.345851793	3.358807946	–6.598175391
299.66	3.337115397	–6.649147321	3.330466250	3.343764545	–6.506515197
300.74	3.325131343	–6.812240140	3.318319103	3.331943583	–6.434316779
			Form II		
286.27	3.493205715	–5.960313653	3.487245401	3.499166029	–7.380540724
288.61	3.464883407	–5.619632539	3.459263774	3.470503039	–7.263344995
290.34	3.44423779	–5.445870568	3.438791920	3.449683661	–7.177914879
291.57	3.429708132	–5.365269067	3.424342863	3.435073401	–7.117792170
293.43	3.407967829	–5.312774901	3.402655054	3.413280604	–7.027832318
295.71	3.381691522	–5.360731583	3.376330791	3.387052254	–6.919102800
297.32	3.363379524	–5.464782370	3.357914741	3.368844306	–6.843329033
299.62	3.33756091	–5.703161717	3.331857749	3.343264072	–6.736493418
300.86	3.323805092	–5.870060209	3.317935032	3.329675152	–6.679572805

10. References

1. Macrae, C. F.; Sovago, I.; Cottrell, S. J.; Galek, P. T. A.; McCabe, P.; Pidcock, E.; Platings, M.; Shields, G. P.; Stevens, J. S.; Towler, M.; et al. Mercury 4.0: from visualization to analysis, design and prediction. *J. Appl. Cryst.* **2020**, *53*, 226-235.

2. Karki, S.; Friscic, T.; Jones, W. Control and Interconversion of Cocrystal Stoichiometry in Grinding: Stepwise Mechanism for the Formation of a Hydrogen-Bonded Cocrystal. *CrystEngComm* **2009**, *11*, 470-481.

3. Feliciano, I. O.; Bernardes, C. E. S.; Minas da Piedade, M. E. Standard Molar Enthalpy of Sublimation of Form I Nicotinamide. *J. Chem. Thermodyn.* **2023**, *182*, 107042-107047.

4. Germann, L. S.; Arhangelskis, M.; Etter, M.; Dinnebier, R. E.; Friščić, T. Challenging the Ostwald Rule of Stages in Mechanochemical Cocrystallisation. *Chem. Sci.* **2020**, *11*, 10092-10100.

5. Khajir, S.; Shayanfar, A.; Martinez, F.; Rahimpour, E.; Jouyban, A. Nicotinamide Solubility in Ethanol + Acetonitrile at Different Temperatures. *Phys. Chem. Res.* **2024**, *12*, 33-45.

6. Ganai, S.; Mahatha, B. C.; Saha, A.; Chakraborty, J.; P., A.; Mukherjee, P.; Shaikh, H.; Mallick, D.; Roy, S. Investigation of the Solubility of Adipic Acid in Binary Water-Organic Solvents H_2O + (DMF, DMSO, and ACN) in the Temperature Range (T = 288.15 K to 313.15 K). *J. Mol. Liq.* **2025**, *432*, 127692.

7. Kiyobayashi, T.; Minas da Piedade, M. E. The Standard Molar Enthalpy of Sublimation of η^5-Bis-pentamethylcyclopentadienyl Iron Measured with an Electrically Calibrated Vacuum-drop Sublimation Microcalorimetric Apparatus. *J. Chem. Thermodyn.* **2001**, *33*, 11-21.

8. Bernardes, C. E. S.; Santos, L. M. N. B. F.; Minas da Piedade, M. E. A New Calorimetric System to Measure Heat Capacities of Solids by the Drop Method. *Meas. Sci. Technol.* **2006**, *17*, 1405-1408.

9. Bernardes, C. E. S. CBCAL: A Data Collection Program for Calorimetry Experiments (Version 3.0). *Zenodo* **2022**, https://doi.org/10.5281/zenodo.6475251.

10. Bernardes, C. E. S. EASY GRAPH II - Making Data Plotting and Analysis Easier (2.1.24.1). *Zenodo* **2024**, https://zenodo.org/doi/10.5281/zenodo.7061809.

11. Irikura, K. K.; Frurip, D. J. Computational Thermochemistry. Prediction and Estimation of Molecular Thermodynamics. In *ACS Symposium Series No. 677*, ACS Publications: Washington, 1998.

12. Becke, A. D. Density-Functional Thermochemistry. III. The Role of Exact Exchange. *J. Chem. Phys.* **1993**, *98*, 5648-5652.

13. Lee, C.; Yang, W.; Parr, R. G. Development of the Colle-Salvetti Correlation-Energy Formula Into a Functional of the Electron Density. *Phys. Rev. B* **1988**, *37*, 785-789.

14. Dunning, T. H. Gaussian Basis Sets for Use in Correlated Molecular Calculations. I. The Atoms Boron Through Neon and Hydrogen. *J. Chem. Phys.* **1989**, *90*, 1007-1023.

15. Kendall, R. A.; Dunning, T. H.; Harrison, R. J. Electron Affinities of the First-Row Atoms Revisited. Systematic Basis Sets and Wave Functions. *J. Chem. Phys.* **1992**, *96*, 6796-6806.

16. Johnson, R. NIST Computational chemistry comparison and benchmark database, Release 22, NIST standard reference database number 101. National Institute of Standards and Technology: 2022.

17. Davies, M.; Thomas, G. H. The Lattice Energies, Infra-Red Spectra, and Possible Cyclization of Some Dicarboxylic Acids. *Trans. Faraday Soc.* **1960**, *56*, 185-192.

18. Albyn, K. C. Extension of the Enthalpy of Sublimation for Adipic Acid to Temperatures Below 80 °C. *J. Chem. Eng. Data* **2001**, *46*, 1415-1416.

19. Bilde, M.; Svenningsson, B.; Mønster, J.; Rosenørn, T. Even−Odd Alternation of Evaporation Rates and Vapor Pressures of C3−C9 Dicarboxylic Acid Aerosols. *Environ. Sci. Technol.* **2003**, *37*, 1371-1378.

20. Cappa, C. D.; Lovejoy, E. R.; Ravishankara, A. R. Determination of Evaporation Rates and Vapor Pressures of Very Low Volatility Compounds: a Study of the C4−C10 and C12 Dicarboxylic Acids. *J. Phys. Chem. A* **2007**, *111*, 3099-3109.

21. Booth, A. M.; Markus, T.; McFiggans, G.; Percival, C. J.; McGillen, M. R.; Topping, D. O. Design and Construction of a Simple Knudsen Effusion Mass Spectrometer (KEMS) System for Vapour Pressure Measurements of Low Volatility Organics. *Atmos. Meas. Tech.* **2009**, *2*, 355-361.

22. Taulelle, P.; Sitja, G.; Pépe, G.; Garcia, E.; Hoff, C.; Veesler, S. Measuring Enthalpy of Sublimation for Active Pharmaceutical Ingredients: Validate Crystal Energy and Predict Crystal Habit. *Cryst. Growth Des.* **2009**, *9*, 4706-4709.

23. Taylor, B. N.; Kuyatt, C. E. *Guidelines for Evaluating and Expressing the Uncertainty of NIST Measurements Results*; NIST Technical Note 1297, 1994.

24. Sheldrick, G. M. *SHELXL-97: Program for the Refinement of Crystal Structure*; University of Göttingen, 1997.

25. Prohaska, T.; Irrgeher, J.; Benefield, J.; Böhlke, J. K.; Chesson, L. A.; Coplen, T. B.; Ding, T.; Dunn, P. J. H.; Gröning, M.; Holden, N. E.; et al. Standard Atomic Weights of the Elements 2021 (IUPAC Technical Report). *Pure Appl. Chem.* **2022**, *94*, 573-600.

26. Miller, J. N. Basic Statistical-Methods for Analytical-Chemistry .Part 2. Calibration and Regression Methods - A Review. *Analyst* **1991**, *116*, 3-14.
